# Minimally-invasive glaucoma surgeries (MIGS) for open angle glaucoma: A systematic review and meta-analysis

**DOI:** 10.1371/journal.pone.0183142

**Published:** 2017-08-29

**Authors:** Carlo Lavia, Laura Dallorto, Milena Maule, Manuela Ceccarelli, Antonio Maria Fea

**Affiliations:** 1 Eye Clinic, Department of Surgical Sciences, University of Turin, Turin, Italy; 2 Department of Medical Sciences, University of Turin and CPO-Piemonte, AOU Città della Salute e della Scienza di Torino, Turin, Italy; 3 SCDU Epidemiologia dei Tumori-CPO Piemonte, AOU Città della Salute e della Scienza di Torino, Turin, Italy; Universita degli Studi di Firenze, ITALY

## Abstract

**Background:**

MIGS have been developed as a surgical alternative for glaucomatous patients.

**Purpose:**

To analyze the change in intraocular pressure (IOP) and glaucoma medications using different MIGS devices (Trabectome, iStent, Excimer Laser Trabeculotomy (ELT), iStent Supra, CyPass, XEN, Hydrus, Fugo Blade, Ab interno canaloplasty, Goniscopy-assisted transluminal trabeculotomy) as a solo procedure or in association with phacoemulsification.

**Methods:**

Randomized control trials (RCT) and non-RCT (non randomized comparative studies, NRS, and before-after studies) were included. Studies with at least one year of follow-up in patients affected by primary open angle glaucoma, pseudoexfoliative glaucoma or pigmentary glaucoma were considered. Risk of Bias assessment was performed using the Cochrane Risk of Bias and the ROBINS-I tools. The main outcome was the effect of MIGS devices compared to medical therapy, cataract surgery, other glaucoma surgeries and other MIGS on both IOP and use of glaucoma medications 12 months after surgery. Outcomes measures were the mean difference in the change of IOP and glaucoma medication compared to baseline at one and two years and all ocular adverse events. The current meta-analysis is registered on PROSPERO (reference n° CRD42016037280).

**Results:**

Over a total of 3,069 studies, nine RCT and 21 case series with a total of 2.928 eyes were included. Main concerns about risk of bias in RCTs were lack of blinding, allocation concealment and attrition bias while in non-RCTs they were represented by patients’ selection, masking of participants and co-intervention management. Limited evidence was found based on both RCTs and non RCTs that compared MIGS surgery with medical therapy or other MIGS. In before-after series, MIGS surgery seemed effective in lowering both IOP and glaucoma drug use. MIGS showed a good safety profile: IOP spikes were the most frequent complications and no cases of infection or BCVA loss due to glaucoma were reported.

**Conclusions:**

Although MIGS seem efficient in the reduction of the IOP and glaucoma medication and show good safety profile, this evidence is mainly derived from non-comparative studies and further, good quality RCTs are warranted.

## Introduction

Glaucoma is the second commonest cause of blindness worldwide [1]. To date, the main treatment for preventing glaucomatous damage consists in lowering intraocular pressure (IOP)[2]. The first ocular hypotensive approach is commonly eye-drop medications, whose instillation is often needed more than once per day. Poor compliance [3–4] and tolerability [5–6] can sometimes lead to treatment failure. Ab externo filtration surgery is still considered the gold standard but it is reserved to progressive disease and may lead to significant complications [7,8]. Minimally-invasive glaucoma surgeries (MIGS) have been developed as safer and less traumatic surgical interventions for patients with mild to moderate glaucoma or who are intolerant to standard medical therapy [9]. According to the commonly accepted definition, MIGS are surgical procedures with an ab-interno approach, minimal trauma with very little or no scleral dissection, minimal or no conjunctival manipulation, good safety profile and rapid recovery [10].

MIGS devices can be divided in: trabecular, suprachoroidal and subconjunctival based [11]. They can be performed in association with cataract surgery or as a solo procedure [12].

The trabecular based devices work by improving trabecular outflow through Schlemm's canal. The suprachoroidal based devices improve the uveoscleral outflow through a connection between the anterior chamber and the suprachoroidal space while the subconjunctival devices create an alternative outflow pathway of the aqueous humor to the subconjunctival space [13,14].

There is a growing interest about MIGS procedures and several clinical studies have been published in the past years. This increase in surgical options should be supported by a clear evidence of their efficacy, to give the surgeon a detailed panorama on the potential surgical options.

However, many clinical studies have been small, nonrandomized, and often lacking appropriate control arms. Moreover, these studies often exhibit great variability in measured outcomes, definition of success/failure and follow-up periods.

The purpose of the current meta-analysis is to analyze available data on MIGS and to summarize and quantify their effect on both intraocular pressure and use of topical glaucoma medications as well as their safety profile.

## Materials and methods

In this research, we adhered to the Preferred Items for Systematic Reviews and Meta-Analyses (PRISMA) guidelines.

### Protocol and registration

The current meta-analysis is registered on PROSPERO (reference n° CRD42016037280) and is available from http://www.crd.york.ac.uk/PROSPERO/display_record.asp?ID=CRD42016037280. During the peer-review process, extensive changes were required to the original protocol.

### Literature search strategy

The construction of search strategies was performed by an expert epidemiologist (MC) using database specific subject headings and keywords. Electronic databases search was performed by two clinicians (LD, CL) and a third member (AF) in case of disagreement. Articles published between January 1, 2000 and December 31, 2016 were included.

Information sources included: MEDLINE Daily and MEDLINE (Ovid), MEDLINE In-Process and Other Non-Indexed Citations, CENTRAL (which contains the Cochrane Eyes and Vision Group Trials Register), EMBASE (Ovid), Latin American and Caribbean Literature on Health Sciences (LILACS), CINAHL (EBSCO), Trip Database and The National Institute for Health and Care Excellence (NICE). The search strategies for MEDLINE are included in the supplementary material (S1 Appendix). These searches were supplemented by hand searching the bibliographies of all the included studies. Grey literature was not considered in this meta-analysis due to excessive lack of essential information that usually affects this type of research.

### Study selection

Study design: Randomized controlled trials (RCT) and non-RCT (non-randomized comparative studies, NRS, and before-after studies) were included. Accepted languages of publication were: English, German, French, Spanish, Portuguese and Italian.

Exclusion criteria:

1. Follow-up shorter than 12 months.
2. Glaucoma types other than primary open angle glaucoma (POAG). pseudoexfoliative (PEX) and pigmentary glaucoma (PG).
3. Number of patients lost at follow-up equal or greater than 15% (in non RCT studies)
4. Any previous glaucoma surgery except laser trabeculoplasty.
5. Studies including patients younger than 18 years.

Interventions:

In accordance with the provided definition of MIGS we included studies regarding:

- Ab interno trabeculotomy, Trabectome device (NeoMedix, Tustin, CA, USA)
- Trabecular Microbypass Stent (iStent, Glaukos, Laguna Hills, CA, USA)
- Schlemm’s Canal Scaffold (Hydrus, Ivantis, Irvine, CA, USA)
- Suprachoroidal Microstent (Cypass Transcend Medical, Menlo Park, CA, USA)
- iStent Supra (iStent, Glaukos, Laguna Hills, CA, USA)
- XEN Subconjuntival Implant gel stent (Aquesys, Aliso Viejo, CA, USA/ Allergan, Irvine, CA, USA)
- Ab interno Canaloplasty (ABIC)
- Excimer Laser Trabeculotomy (ELT, Glautec AG, Nurnberg, Germany)
- Gonioscopy-Assisted Transluminal Trabeculotomy (GATT)
- Fugo plasma blade (MediSurg Research and Management Corp., Norristown, PA, USA)

Studies about MIGS as a solo procedure or combined with cataract surgery were considered.

Screening of titles and abstracts was carried out and not pertinent articles were rejected. Full texts of residual articles were evaluated independently for eligibility. The process was made according to the PRISMA flow diagram. Duplicates were removed using EPPI reviewer (by EPPI-Center, Social Science Research Unit, the Institute of Education, the University of London, UK).

### Data collection and risk of bias assessment

The main outcome of this meta-analysis was the effect of MIGS devices compared to medical therapy, cataract surgery, other glaucoma surgeries and other MIGS on the change in both IOP and use of glaucoma medications 12 months after surgery. Outcomes were analyzed separately for every MIGS, as well as for the solo and the combined procedures.

Secondary outcomes were:

- the effect of MIGS on the change in IOP and glaucoma medications between baseline and 12 and 24 months
- surgery-related adverse events.

The measure of effect was the mean change in IOP (mmHg) and the mean change in number of antiglaucoma medications, since change is expected to be less dependent of differences in baseline values.

Data were collected from each study independently by two reviewers (LD, CL). In order to obtain or confirm missing or uncertain data from investigators, corresponding Authors were contacted twice by email. In case of impossibility to obtain missing/incomplete data from corresponding Authors, data were extrapolated from figures/graphics when available. Given the potential inaccuracy of these data, outcomes were also analyzed separately excluding all inferred-results-studies, as a sensitivity analysis.

The quality assessment was evaluated with the latest version of the risk of Bias tool recommended in the Cochrane handbook for systematic review of Interventions (Chapter 8, Higgins 2011) [15] for RCT and the ROBINS-I checklist for non-RCT [16]. RCT studies were judged for the selection bias, performance bias, detection bias, attrition bias, reporting bias and other sources of bias.

Non RCT studies were judged for confounding bias, selection bias, bias in classification of interventions, bias in deviation from intended interventions, bias due to missing data, bias in measurement of outcome and bias in selection of the reported results. Results from ROBINS-I were separately reported for NRS and before-after studies, considering that the use of ROBINS-I in before-after studies is exploratory and has not been validated yet.

Studies were not excluded a priori based on quality reporting assessment.

### Data synthesis and analysis

Data were analyzed from March to May 2017. Statistical analysis was performed using STATA (software version 13.1, STATA corporation, College Station, TX, USA). The random effect method was used to perform statistical analysis.

We presented mean and standard error (SE) of the IOP and number of glaucoma medication at baseline and endpoint were used to compute their mean reduction and mean and standard deviation of IOP percentage of reduction (IOPR %, SD-IOPR%):

$$\text{IOPR}={\text{IOP}}_{\text{baseline}}-{\text{IOP}}_{\text{endpoint}}$$

$$\text{IOPR}\%=\frac{\text{IOPR}}{{\text{IOP}}_{\text{baseline}}}$$

$$\text{SD-IOPR}\%=\frac{\sqrt{{\left({\text{SE}}_{\text{baseline}}\right)}^{2}+{\left({\text{SE}}_{\text{endpoint}}\right)}^{2}}}{{\text{IOP}}_{\text{baseline}}}*\sqrt{n}$$

(n: number of patients at the baseline).

When a meta-analysis was possible, summary effect measures were reported as weighted mean difference [17,18]; when the SD of the mean change was not available, the following formula was used to imputed it:

$${SD}_{CHANGE}=\sqrt{{SD}_{baseline}^{2}+{SD}_{final}^{2}-(2*Corr*{SD}_{baseline}*{SD}_{final})}$$

A correlation value (Corr) of 0.3 was used as it was the median value of the available SD values.

To investigate heterogeneity among studies, I^2^ statistics were computed. Large statistical heterogeneity was generally found, due to the large precision of estimates in this type of matched studies and to the use of continuous outcomes [19]. We had planned to use funnel plots were generated and both Egger’s [20] and Begg’s [21] tests to assess the risk of publication bias but the limited number of studies did not allow this type of testing.

## Results

### Study selection

**Fig 1:**
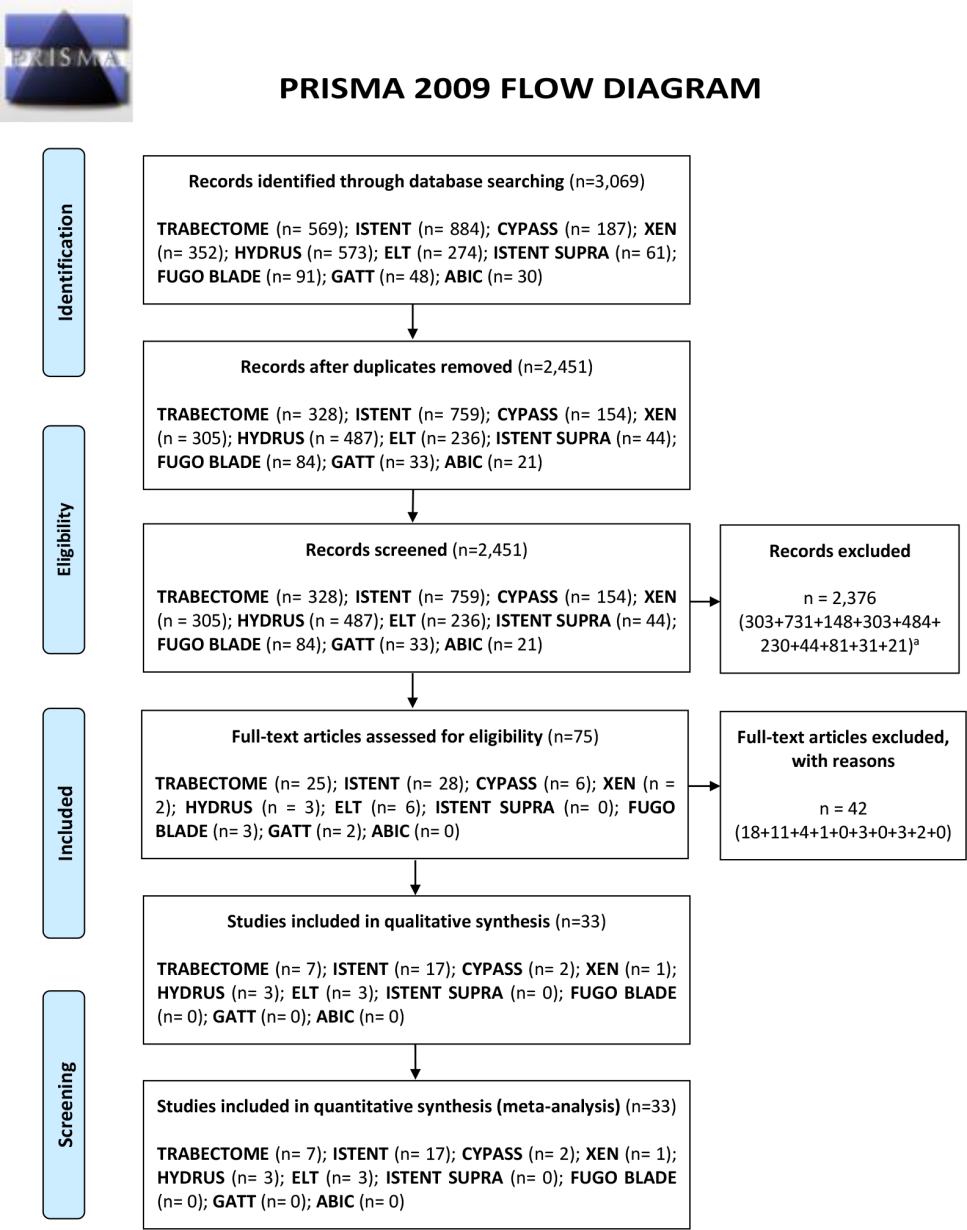
PRISMA flow diagram ^a^ = number of studies excluded for device respected the same order as the main boxes.

A total of 3,069 studied were screened using the described search strategy. At the end of the selection process, 30 studies were identified [22–51]. 3 out of 30 studies compared Trabectome with iStent surgery[22,23,25] while 2 out of 30 studies compared Trabectome combined and solo procedures[26,27]. Analyzed studies included 9 RCTs (Trabectome = 0, iStent = 6, CyPass = 1, Aquesys = 0, Hydrus = 1 and ELT = 1) and 21 non-RCTs (Trabectome = 7 (7 NRS), iStent = 11 (4 NRS, 7 before-after), CyPass = 1 (before-after), XEN = 1 (before-after), Hydrus = 2 (2 NRS) and ELT = 2 (1 NRS, 1 before-after). The RCTs included 850 eyes while 2,078 eyes were included in the non RCTs studies (1,598 eyes in NRS, 480 eyes in before-after studies). No studies regarding iStent Supra, ABIC, GATT and Fugo Blade met the eligibility criteria. Overall there were 20 studies about combined procedures (1521 eyes) and 15 about solo procedures (1407 eyes). Further details on included studies are given in the Prisma flow diagram (Fig 1).

Excluded studies on the basis of full-text are referenced in the supplementary material (S2 Appendix).

### Study characteristics

Baseline characteristics of included studies are reported in Table 1.

**Table 1:** Baseline characteristics of included studies.

Device	Author, Year	Study Design	Comparator	Eyes (n)	Age (Years) Mean ± SD	Sex M/F (%)	POAG (%)	PEX (%)	PIGM (%)	Follow-up (months)
**TRABECTOME**										
COMBINED										
	GONNERMANN^a^, 2016^22^	NRS	ISTENT COMBINED	27	73.8 ± 7.8	48/52	70	30	0	12
	KHAN, 2015^23^	NRS	ISTENT COMBINED	52	76.1 ± 12.1	44/56	96	2	2	12
	KLAMANN, 2013^24^	NRS	TRABECULAR ASPIRATION COMBINED	27	73.4 ± 10.8	56/44	0	100	0	12
	KURJI, 2016^25^	NRS	ISTENT COMBINED	36	72.4 ± 9.6	53/47	56	44	0	12
	PAHLITZSCH, 2015 (POAG)^26^	NRS	TRABECTOME SOLO	130	NR	NR	100	0	0	12
	PAHLITZSCH, 2015 (PEX)^26^	NRS	TRABECTOME SOLO	54	NR	NR	0	100	0	12
	TING, 2012 (POAG)^27^	NRS	TRABECTOME SOLO	263	74 ± 9	40/59	100	0	0	12
	TING, 2012 (PEX)^27^	NRS	TRABECTOME SOLO	45	78 ± 7	24/73	0	100	0	12
SOLO										
	PAHLITZSCH, 2015 (POAG)^26^	NRS	TRABECTOME COMBINED	72	NR	NR	100	0	0	12
	PAHLITZSCH, 2015 (PEX)^26^	NRS	TRABECTOME COMBINED	40	NR	NR	0	100	0	12
	PAHLITZSCH, 2016 (POAG)^28^	NRS	MODIFIED GONIOTOMY	119	73.9 ± 9.6	NR	100	0	0	12
	PAHLITZSCH, 2016 (PEX)^28^	NRS	MODIFIED GONIOTOMY	27	75.2 ± 8.0	NR	0	100	0	12
	TING, 2012 (POAG)^27^	NRS	TRABECTOME COMBINED	450	68 ± 15	40/57	100	0	0	12
	TING, 2012 (PEX)^27^	NRS	TRABECTOME COMBINED	67	78 ± 7	24/73	0	100	0	12
**ISTENT**										
COMBINED										
1 STENT										
	ARRIOLA-VILLALOBOS, 2012^29^	BEF-AFT STUDY	N/A	19	74.6 ± 8.4	47/53	79	10.5	10.5	60
	CRAVEN^b^, 2012^30^	RCT	PHACOEMULSIFICATION ALONE	117	74 ± 8	39/61	91	6	3	24
	FEA, 2015^31^	RCT	PHACOEMULSIFICATION ALONE	10	63.9^c^ ± 3.2^c^	30/70^c^	100^c^	0^c^	0^c^	48
	SPIEGEL, 2009^32^	BEF-AFT STUDY	N/A	48	76.2 ± 6.7	38/62	74^d^	17^d^	9^d^	12
2 STENT										
	ARRIOLA-VILLALOBOS^e^, 2016 (INJECT)^33^	BEF-AFT STUDY	N/A	20	75.1 ± 8.6	45/55	40–40 OHT	20	0	60
	BELOVAY^f^, 2012^34^	NRS	PHACOEMULSIFICATION+3 ISTENT	28	78.8 ± 7	27/73	75	25	0	12
	FERNÁNDEZ-BARRIENTOS, 2010^35^	RCT	PHACOEMULSIFICATION ALONE	17	75.2 ± 7.2	35/65	88.2–11.8 OHT	0	0	12
	GONNERMANN^a^, 2016 (INJECT)^22^	NRS	TRABECTOME COMBINED	27	73.8 ± 7.8	48/52	70	30	0	12
	KHAN, 2015^23^	NRS	TRABECTOME COMBINED	49	77.5 ± 11.9	41/59	78	22	0	12
	KURJI, 2016^25^	NRS	TRABECTOME COMBINED	34	75.0 ± 10.3	44/56	56	44	0	12
SOLO										
1 STENT										
	KATZ, 2015^36^	RCT	2 AND 3 ISTENT SOLO	38	68.1 ± 9.1	71/29	100	0	0	18
2 STENT										
	AHMED, 2014^37^	BEF-AFT STUDY	N/A	39	62.8 ± 12.6	54/46	NR	NR	NR	18
	DONNENFELD, 2015^38^	BEF-AFTER STUDY	N/A	39	66.7 ± 10.0	56/44	NR	NR	NR	36
	FEA, 2014^39^ (INJECT)	RCT	2 MEDICATIONS	94	64.5 ± 10.3	39/61	NR	NR	NR	12
	KATZ, 2015^36^	RCT	1 AND 3 ISTENT SOLO	41	67.8 ± 9.3	46/54	97.6	2.4	0	18
	LINDSTROM, 2016^40^ (INJECT)	BEF-AFT STUDY	N/A	57	65.3 ± 9.0	53/47	100	0	0	18
	VOLD, 2016^41^	RCT	MEDICATION	54	64.5 ± 11.1	46/54	100	0	0	36
	VOSKANYAN, 2014^42^ (INJECT)	BEF-AFT STUDY	N/A	99	66.4 ± 10.9	43/57	97	3	0	12
3 STENT										
	KATZ, 2015^36^	RCT	1 AND 2 ISTENT SOLO	40	60.9 ± 8.1	48/52	100	0	0	18
**CYPASS**										
COMBINED										
	VOLD, 2016^43^	RCT	PHACOEMULSIFICATION ALONE	374	70.0 ± 8.0	47/53	100	0	0	24
SOLO										
	GARCÍA-FEIJOO, 2015^44^	BEF-AFT STUDY	N/A	65	68.3 ± 10.5	31/69	100	0	0	12
**XEN**										
COMBINED										
	PEREZ-TORREGROSA, 2016^45^	BEF-AFT STUDY	N/A	30	76 ± 5.9	28/72	100^c^	0^c^	0^c^	12
**HYDRUS**										
COMBINED										
	PFEIFFER, 2015^46^	RCT	PHACOEMULIFICATION ALONE	50	72.8 ± 6.6	40/60	90	10	0	24
SOLO										
	GANDOLFI, 2016^47^	NRS	CANALOPLASTY AB EXTERNO	21	NR	71/29	57.1	33.3	9.5	24
	FEA, 2016^48^	NRS	SLT	31	70.8 ± 11.8	58/42	100	0	0	12
**ELT**										
COMBINED										
	TÖTEBERG-HARMS, 2013^49^	BEF-AFT STUDY	N/A	64	76.5 ± 9.4	34/66	33–3 NTG—6 OHT	58	0	12
SOLO										
	BABIGHIAN, 2006^50^	NRS	MEDICAL THERAPY (FELLOW EYE)	21	58 ± 8.9	43/57	100	0	0	24
	BABIGHIAN, 2010^51^	RCT	SLT	15	65.2 ± 4.6	40/60	100	0	0	24

Number rounded to the first decimal digit. If data discordance between text and tables, data from the text was chosen. SD = standard deviation. NRS = non-randomized study of intervention. RCT = randomized controlled trial. Bef-aft study = before-after study. POAG = primary open angle glaucoma. Pex = pseudoexfoliative glaucoma. Pigm = pigmentary glaucoma. NTG = normal tension glaucoma. OHT = ocular hypertension. NR = not reported. N/A = not applicable

^a^ = demographic data from 27 patients, IOP and medication data from 25 eyes of 25 patients (excluded from the study patients with secondary glaucoma surgery).

^b^ = demographic and baseline data obtained from Samuelson 2011^52^.

^c^ = reported by corresponding author.

^d^ = OAG subtypes data from intention-to-treat(ITT) population (58 patients), demographic and baseline data from per-protocol(PP) population (48 patients).

^e^ = 3 patients received only 1 iStent. Demographic data from Arriola-Villalobos 2013^53^.

^f^ = 3-iStent group data excluded because of 8% mixed mechanism

Tables 2 and 3 reported IOP and number of medications at baseline and 1-year follow-up in different type of studies, together with their percent reduction.

**Table 2:** IOP and antiglaucoma medication values at baseline and one year in RCT and NRS.

Author, Year	Study Design	Group 1	Group 2	GROUP 1							GROUP 2						
				Eyes (n)	IOP Bas (mmHg) Mean ± SD	IOP 1 year (mmHg) Mean ± SD	IOP Reduction (%) Mean ± SD	Med Bas (n) Mean ± SD	Med 1 year (n) Mean ± SD	Med Reduction (n)	Eyes (n)	IOP Bas (mmHg) Mean ± SD	IOP 1 year (mmHg) Mean ± SD	IOP Reduction (%) Mean ± SD	Med Bas (n) Mean ± SD	Med 1 year (n) Mean ± SD	Med Reduction (n)
GONNERMANN^a^, 2016^22^	NRS	TRABECTOME COMBINED	ISTENT COMBINED	25	22.3 ± 3.7	15.6 ± 3.6	30.0 ± 23.2	2.1 ± 1.1	1.4 ± 1.3	0.64	25	21.3 ± 4.1	14 ± 2.3	34.3 ± 22.1	2.0 ± 0.9	1.3 ± 1.2	0.76
KHAN, 2015^23^	NRS	TRABECTOME COMBINED	ISTENT COMBINED	52	20.6 ± 6.8	17.3 ± 6.5^b^	16.0 ± 45.7	2.9 ± 1.1	2.2 ± 1.4	0.75	49	19.6 ± 5.3	14.3 ± 3.1	27.0 ± 31.3	2.9 ± 0.9	1.2 ± 1.3	1.64
KLAMANN, 2013^24^	NRS	TRABECTOME COMBINED	TRABECULAR ASPIRATION COMBINED	27	23.4 ± 5.9	14.1 ± 2.3	39.7 ± 26.9	2.3 ± 1.0	2 ± 1	0.29	28	22.2 ± 6.3	17.1 ± 4.0	23. 0 ± 33.7	2.2 ± 1.1	2.3 ± 1.3	- 0.08
KURJI, 2016^25^	NRS	TRABECTOME COMBINED	ISTENT COMBINED	36	20.9 ± 5.1	15.8 ± 3.8^c^	24.3 ± 30.8	2.3 ± 1.3	1.8 ± 1.3^c^	0.49	34	17.5 ± 4.9	13.7 ± 4.4^c^	22.0 ± 38.4	2.2 ± 1.2	1.9 ± 1.2^c^	0.26
PAHLITZSCH, 2015 (POAG)^26^	NRS	TRABECTOME COMBINED	TRABECTOME SOLO	130	19.2 ± 4	11.8 ± 3.1	38.5 ± 26.4	2.3 ± 0.8	2.3 ± 1.4	0	72	19.8 ± 5.9	14.8 ± 3.2	25.3 ± 33.9	2.6 ± 0.8	2.1 ± 1.2	0.50
PAHLITZSCH, 2015 (PEX)^26^	NRS	TRABECTOME COMBINED	TRABECTOME SOLO	54	23.2 ± 9.2	12.6 ± 1.1	45.7 ± 39.9	2.2 ± 1.1	1.4 ± 0.8	0.80	40	23.7 ± 9.5	14.0 ± 3.3	40.9 ± 42.4	2.5 ± 0.9	2.4 ± 1.2	0.10
TING, 2012 (POAG)^27^	NRS	TRABECTOME COMBINED	TRABECTOME SOLO	263	19.9 ± 5.4	15.6 ± 3.2	21.6 ± 31.8	2.4 ± 1.1	1.7 ± 1.3	0.75	450	25.5 ± 7.9	16.8 ± 3.9	34.1 ± 36.3	2.7 ± 1.3	2.2 ± 1.3	0.57
TING, 2012 (PEX)^27^	NRS	TRABECTOME COMBINED	TRABECTOME SOLO	45	21.7 ± 8.4	14.2 ± 3.1	34.6 ± 41.4	2.5 ± 1.0	1.6 ± 1.3	0.96	67	29.0 ± 7.5	16.1 ± 4.0	44.5 ± 30.2	3.1 ± 1.2	2.2 ± 1.3	0.88
PAHLITZSCH, 2016 (POAG)^28^	NRS	TRABECTOME SOLO	MODIFIED GONIOTOMY	119	18.4 ± 5.3	13.8 ± 3.6	24.9 ± 34.8	2.4 ± 0.9	2.2 ± 0.9	0.25	68	20.2 ± 6.2	14.3 ± 5.7	28.8 ± 41.6	2.6 ± 1.0	1.5 ± 1.2	1.10
PAHLITZSCH, 2016 (PEX)^28^	NRS	TRABECTOME SOLO	MODIFIED GONIOTOMY	27	22.0 ± 9.8	12.3 ± 2.7	44.0 ± 46.2	2.4 ± 1.0	1.8 ± 0.8	0.56	22	20.9 ± 6.1	14.1 ± 6.4	32.3 ± 42.1	2.6 ± 1.0	1.9 ± 1.5	0.67
CRAVEN^d^, 2012^30^	RCT	1 ISTENT COMBINED	PHACO ALONE	98	18.6 ± 3.4	17 ± 2.8	8.6 ± 23.7	1.6 ± 0.8	0.2 ± 0.6	1.40	101	17.9 ± 3.0	17.0 ± 3.1	5.0 ± 24.1	1.5 ± 0.6	0.4 ± 0.7	1.10
FEA, 2015^31^	RCT	1 ISTENT COMBINED	PHACO ALONE	10	17.8 ± 2.7	14.7 ± 1.3	17.4 ± 16.8	1.9 ± 0.9	0.4 ± 0.7	1.50	24	16.7 ± 3.0	15.6 ± 1.1	6.6 ± 19.3	1.8 ± 0.7	1.0 ± 1.0	0.80
FERNÁNDEZ-BARRIENTOS, 2010^35^	RCT	2 ISTENT COMBINED	PHACO ALONE	17	24.2^e^ ± 1.8^e^	17.6 ± 2.8	27.3 ± 13.8	1.1 ± 0.5	0 ± 0	1.10	16	23.6 ± 1.5	19.8 ± 2.3	16.1 ± 11.6	1.2 ± 0.7	0.7 ± 1.0	0.50
KATZ, 2015^36^	RCT	1 ISTENT SOLO	ISTENT (2) SOLO	38	19.8 ± 1.3	14.4 ± 1.2	27.3 ± 9.0	1.7 ± 0.6	0.1 ± 0.3	1.60	41	20.1 ± 1.6	12.8 ± 1.4	36.3 ± 10.6	1.8 ± 0.5	0.1 ± 0.4	1.64
FEA, 2014^39^	RCT	2 ISTENT (INJECT) SOLO	MEDICATION	94	21.1 ± 1.7	13 ± 2.3	38.4 ± 13.6	1.0^f^ ± 0	0.04 ± 0.2^g^	0.96	98	20.7 ± 1.7	13.2 ± 2.0	36.2 ± 13.0	1.0^f^ ± 0	2.0^f^ ± 0	N/A
VOLD^h^, 2016^41^	RCT	2 ISTENT SOLO	MEDICATION	54	25.5 ± 2.5	13.7 ± 1.9^c^	46.3 ± 12.4	0	0.09 ± 0.4^c^	N/A	47	25.1 ± 4.6	13.9 ± 1.7	44.6 ± 19.5	0	1.0^f^ ± 0	N/A
VOLD^i^, 2016^43^	RCT	CYPASS COMBINED	PHACO ALONE	374	24.4^e^ ± 2.8^e^	16.5^g^^,^^e^ ± 4.3^g^^,^^e^	32.4 ± 21.1	1.4 ± 0.9	0.2 ± 0.6	1.20	131	24.5^e^ ± 3.0^e^	18.3^g^^,^^e^ ± 4.5^g^^,^^e^	25.3 ± 22.1	1.3 ± 1.0	0.7 ± 0.9	0.60
PFEIFFER, 2015^46^	RCT	HYDRUS COMBINED	PHACO ALONE	50	18.9 ± 3.3	16.1^g^ ± 3^g^	14.8 ± 23.7	2 ± 1	0.5^g^ ± 1.1^g^	1.50	50	18.6 ± 3.8	16.0^g^ ± 2.8^g^	14.0 ± 25.5	2.0 ± 1.0	0.8^g^ ± 1.1^g^	1.20
FEA, 2016^48^	NRS	HYDRUS SOLO	SLT	31	23.1 ± 5.1	16.5 ± 2.6	28.6 ± 24.8	2.3 ± 0.8	0.9 ± 1.0	1.40	25	23.2 ± 2.2	15.9 ± 2.5	31.4 ± 14.2	2.5 ± 0.9	2.0 ± 0.9	0.48
BABIGHIAN, 2006^50^	NRS	ELT SOLO	MEDICATION (FELLOW EYE)	21	24.8 ± 2.0	16.2 ± 2.1^c^	34.7 ± 11.7	2.2 ± 0.6	NR	N/A	21	21.6 ± 1.6	21.0^c^ ± 2.0^c^	2.8 ± 11.9	NR	NR	N/A
BABIGHIAN, 2010^51^	RCT	ELT SOLO	SLT	15	25.0 ± 1.9	16.0^c^ ± 2.2^c^	36.0 ± 11.6	2.3 ± 0.6	NR	N/A	15	23.9 ± 0.9	19.0^c^ ± 1.8^c^	20.5 ± 8.4	2.2 ± 0.7	NR	N/A

Number rounded to the first decimal digit, (second decimal for medication reduction). If data discordance between text and tables, data from the text was chosen. SD = standard deviation. NRS = non randomized study of intervention. RCT = randomized controlled trial. POAG = primary open angle glaucoma. Pex = pseudoexfoliative glaucoma. IOP = intraocular pressure. NR = not reported. N/A = not applicable

^a^ = demographic data from 27 patients, IOP and medication data from 25 eyes of 25 patients (excluded patients with secondary glaucoma surgery).

^b^ = LOCF (last observation carried forward).

^c^ = data inferred from graphics/figures.

^d^ = demographic and baseline data obtained from Samuelson 2011^52^; outcomes obtained from Craven 2012^30^ (consistent cohort of 98 eyes).

^e^ = wash-out iop.

^f^ = medication use per protocol.

^g^ = reported by corresponding author.

^h^ = naïve glaucoma patients.

^i^ = 1-year IOP from PP population (332 eyes), all other data from ITT population (374 eyes)

**Table 3:** IOP and antiglaucoma medication at baseline and one year in before-after studies.

Author, Year	Device	Eyes (n)	IOP Baseline (mmHg) Mean ± SD	IOP 1 year (mmHg) Mean ± SD	IOP Reduction (%) Mean ± SD	Medication Baseline (n) Mean ± SD	Medication 1 year (n) Mean ± SD	Medication Reduction (n)
ARRIOLA-VILLALOBOS, 2012^29^	1 ISTENT COMBINED	19	19.4 ± 1.9	17.3 ± 3.2^a^	11.0 ± 19.2	1.3 ± 0.5	0.2 ± 0.4	1.15
SPIEGEL^b^, 2009^32^	1 ISTENT COMBINED	48	21.7 ± 4.0	17.4 ± 3.0	19.8 ± 23.5	1.6 ± 0.8	0.4 ± 0.6	1.20
ARRIOLA-VILLALOBOS^c^, 2016 ^33^	2 ISTENT (INJECT) COMBINED	20	20.0 ± 3.7	16.8 ± 2.2	16.0 ± 21.7	1.3 ± 0.7	0.3 ± 0.6	1.00
BELOVAY^d^, 2012^34^	2 ISTENT COMBINED	28	17.3 ± 4.0	13.8 ± 3.4^a^	20.2 ± 30.4	2.8 ± 0.8	1.0 ± 1.3^a^	1.80
AHMED, 2014^37^	2 ISTENT SOLO	39	22.2 ± 2	13.0 ± 2.4	41.4 ± 14.1	2.0 ^e^ ± 0	1.0 ± 0^e^	1.00^e^
DONNENFELD, 2015^38^	2 ISTENT SOLO	39	20.6 ± 2	13.5^f^ ± 1.8^f^	34.5 ± 13.3	1.0 ^e^ ± 0	NR	N/A
LINDSTROM, 2016^40^	2 ISTENT (INJECT) SOLO	57	19.5 ± 1.5	14.2 ± 1.9	27.2 ± 12.4	1^e^ ± 0	0	1.00
VOSKANYAN, 2014^42^	2 ISTENT (INJECT) SOLO	99	22.1 ± 3.3	15.7 ± 3.7	29.0 ± 23.2	2.2 ± 0.4	NR	N/A
GARCÍA-FEIJOO, 2015^44^	CYPASS SOLO	65	24.5 ± 2.8	16.4 ± 5.5	33.1 ± 27.0	2.2 ± 1.1	1.4 ± 1.3	0.80
PEREZ-TORREGROSA, 2016^45^	XEN COMBINED	30	21.2 ± 3.4	15.0 ± 2.5	29.1 ± 19.8	3.1 ± 0.7	0.2 ± 0.7	2.90
TÖTEBERG-HARMS, 2013^49^	ELT COMBINED	64	19.8 ± 5.3	15.2 ± 4.4	23.2 ± 34.8	2.4 ± 1.1	1.5 ± 1.4	0.90

Number rounded to the first decimal digit, (second decimal for medication reduction). If data discordance between text and tables, data from the text was chosen. SD = standard deviation. IOP = intraocular pressure. NR = not reported. N/A = not applicable

^a^ = data inferred from graphics/figures.

^b^ = baseline data from per-protocol(PP) population (48 patients), 12-months IOP and medication on 42 patients (some patients excluded by the study due to secondary surgery)

^c^ = 3 patients received only 1 iStent. SD 1 year from Arriola-Villalobos 2013^53^

^d^ = considered as a before-after study as the 3-iStent group was excluded due to 8% mixed mechanism.

^e^ = medication use per protocol.

^f^ = calculated on 92.3% patients without medication.

In two studies [30,33] missing data were taken from previous papers by the same authors [52,53], being the former the long-term follow-up studies.

Baseline characteristics of included patients who were given indication for MIGS therapy were highly variable in terms of glaucoma severity, IOP values (e.g medicated and unmedicated) and number of glaucoma medications. The majority of studies reported visual field and clinical parameters to describe the baseline glaucoma severity of patients. In all the studies but three mild to moderate glaucoma were included, while in three papers advanced glaucoma [25,34] (Mean Deviation ≥ -12 dB) and need for filtrating surgery were baseline findings [44].

Medicated IOP had to be between 18 and 30 mmHg in some studies [e.g. 36, 37, 38, 40], even though different thresholds were chosen by other authors (i.e. 14–30 mmHg [33], 17–31 mmHg [35], 21–35 mmHg [44], > 18 mmHg [25]). Some inclusion criteria considered as a cutoff value the target IOP [e.g. 22,34].

Washed-out IOP values were even variable across studies, being for example 22–32 [33] or 22–38 mmHg [36, 37,38,39,40], but not all studies contemplate wash-out, mainly due to ethical implications.

Only one study [41] included naïve patients, three studies [38,39,40] in the iStent as a solo procedure group included patients on one hypotensive medication and POAG subjects on 2 topical therapy were considered eligible in the study by Ahmed [37].

Main outcomes at follow-up visit over one year are reported in Table 4.

**Table 4:** Pre- and post-operative intraocular pressure and glaucoma medications at last follow-up visit of included studies.

Device	Author, Year	Eyes (N)	Postoperative Follow-up (months)	IOP Baseline (mmHg) Mean ± SD	IOP Postoperative (mmHg) mean ± SD	IOP reduction (%) mean ± SD	Medication baseline (N) Mean ± SD	Medication postoperative (N) Mean ± SD	Medication reduction (N)
**ISTENT**									
COMBINED									
1 STENT									
	ARRIOLA-VILLALOBOS, 2012^29^	19	24	19.4 ± 1.9	16.1 ± 3.0^a^	17.0 ± 18.4	1.3 ± 0.5	0.3 ± 0.5	1.00
	ARRIOLA-VILLALOBOS, 2012^29^	19	36	19.4 ± 1.9	15.9 ± 3.6^a^	17.9 ± 20.8	1.3 ± 0.5	0.6 ± 0.6	0.76
	ARRIOLA-VILLALOBOS, 2012^29^	16	48	19.4 ± 1.9	16.5 ± 3.6^a^	15.2 ± 22.4	1.3 ± 0.5	0.5 ± 0.6	0.82
	CRAVEN ^b^, 2012^30^	117	24	18.6 ±3.4	17.1 ± 2.9	8.1 ± 24.0	1.6 ± 0.8	0.3 ± 0.6	1.30
	FEA, 2015^31^	10	48	17.8 ± 2.7	15.9 ± 2.3	10.7 ± 19.9	1.9 ± 0.9	0.5 ± 0.8	1.40
2 ISTENT									
	ARRIOLA-VILLALOBOS, 2016 (INJECT)^33^	19	24	20.0 ± 3.7	17.0 ± 2.3^c^	14.8 ± 22.0	1.3 ± 0.7	0.5 ± 0.6^c^	0.83
	ARRIOLA-VILLALOBOS, 2016 (INJECT) ^33^	15	36	20.0 ± 3.7	17.1 ± 2.2^c^	14.4 ± 22.5	1.3 ± 0.7	0.7 ± 0.7^c^	0.57
	ARRIOLA-VILLALOBOS, 2016 (INJECT) ^33^	12	48	20.0 ± 3.7	17.5 ± 3.5^c^	12.5 ± 29.2	1.3 ± 0.7	0.7 ± 0.8^c^	0.57
	ARRIOLA-VILLALOBOS, 2016 (INJECT) ^33^	11	60	20.0 ± 3.7	16.2 ± 2.3	18.9 ± 24.2	1.3 ± 0.7	1.1± 0.8^c^	0.21
SOLO									
1 ISTENT									
	KATZ, 2015^36^	36	18	19.8 ± 1.3	15.6 ± 1.5	21.2 ± 10.2	1.7 ± 0.6	0.2 ± 0.4	1.52
2 STENT									
	AHMED, 2014^37^	39	18	22.2 ± 2	11.8 ± 2.1	46.9 ± 13.1	2 ± 0^d^	1 ± 0^d^	1.0
	DONNENFELD, 2015^38^	39	24	20.6 ± 2	13.5^e^ ± 2.1^e^	34.5 ± 14.4	1 ± 0^d^	NR	N/A
	DONNENFELD, 2015^38^	30	36	20.6 ± 2	15.2^f^ ± 2.1^f^	26.2 ± 15.5	1 ± 0^d^	NR	N/A
	KATZ, 2015^36^	41	18	20.1 ± 1.6	13.8 ± 1.3	31.3 ± 10.3	1.8 ± 0.5	0.1 ± 0.4	1.64
	LINDSTROM, 2016 (INJECT)^40^	57	18	19.5 ± 1.5	14.4 ± 2.1	26.2 ± 13.2	1 ± 0^d^	0	1.0
	VOLD, 2016^41^	54	24	25.5 ± 2.5	13.8 ± 1.4^a^	45.9 ± 11.3	0^g^	0.13 ± 0.44^a^	N/A
	VOLD, 2016^41^	34	36	25.5 ± 2.5	14.6 ± 2.4^a^	42.8 ± 15.4	0^g^	0.21 ± 0.54^a^	N/A
3 ISTENT									
	KATZ, 2015^36^	38	18	20.4 ± 1.8	12.1 ± 1.2	40.7 ± 10.7	1.5 ± 0.7	0.1 ± 0.3	1.43
**CYPASS**									
COMBO									
	VOLD^h^, 2016^43^	374	24	24.4^i^ ± 2.8^i^	17.0^i^ ± 3.4^i^	30.3 ± 18.7	1.4 ± 0.9	0.2 ± 0.6	1.20
**HYDRUS**									
COMBINED									
	PFEIFFER, 2015^46^	50	24	18.9 ± 3.3	16.5^a^ ± 2.9^a^	12.7 ± 23.7	2.0 ± 1.0	0.5 ± 1.0	1.50
SOLO									
	GANDOLFI, 2016^47^	21	24	24.0 ± 6.0	15.0 ± 3.0	37.5 ± 28.0	3.1 ± 0.6	0.9 ± 0.9	2.20
**ELT**									
SOLO									
	BABIGHIAN, 2006^50^	21	24	24.8 ± 2.0	16.9 ± 2.1	31.9 ± 11.7	2.2 ± 0.6	0.7 ± 0.8	1.53
	BABIGHIAN, 2010^51^	15	24	25.0 ± 1.9	17.6 ± 2.2	29.6 ± 11.6	2.3 ± 0.6	0.7 ± 0.8	1.54

Data at follow-up over 1 year. SD = standard deviation. IOP = intraocular pressure. NR = not reported.

^a^ = data inferred from graphics/figures.

^b^ = demographic and baseline data obtained from Samuelson 2011^52^. Outcomes obtained from Craven 2012^30^ (consistent cohort of 98 eyes).

^c^ = reported by corresponding author.

^d^ = medication use per protocol.

^e^ = calculated on 92.3% patients without medications.

^f^ = calculated on 93.3% patients without medications.

^g^ = naïve glaucoma patients.

^h^ = 2-year IOP on PP population (332 eyes), all other data on ITT population (374 eyes).

^i^ = wash-out IOP

### Risk of bias

#### RCT

Risk of bias assessment for individual RCTs are reported in S1 Table, S1 and S2 Figs. In RCT studies greater risks were found in detection and in the attrition bias. Three studies were considered at high risk of attrition bias [31] and detection bias [36,41]. Unclear risk of allocation concealment was observed in almost all studies, mainly due to incomplete description. No masking of patients, surgeons and outcome assessors seems to be the main concern among the analyzed studies, representing the most serious bias and potentially confounding the outcomes.

Funding by device industry or authors affiliations are often declared (7 out of 9 studies), details are reported in S4 Table.

#### NRS

Risk of bias assessment for NRS are reported in S2 Table and S3 Fig.

A serious risk of confounding was observed in all studies except one that applied a propensity score based on relevant baseline variables [48]. All other NRS were at serious risk of confounding bias due to a lack of baseline IOP and medication adjustment between groups. Some studies recruited consecutive patients and were judged at low risk of bias in selection of participants; other authors excluded patients who did not complete the follow-up: these papers were therefore judged at serious risk [22,23,28,47]. As MIGS and cataract surgery are well-defined once only interventions, misclassification of interventions was unlikely. Glaucoma therapy was the main cointervention, potentially causing serious performance bias. Probably due to the clinical-setting of most NRS, glaucoma medications were often reintroduced or discontinued in the follow-up at clinicians’ discretion (judged as serious risk). If glaucoma therapy was prescribed basing on target IOP, studies were considered at moderate risk [24,28,48]. In our review, studies presenting with more than 15% of patients lost at one-year follow-up were excluded to avoid as much as possible a bias due to missing data. Thus, all included studies were judged at low risk. Due to a lack in masking strategy, outcome assessment was judged at moderate risk of bias in all studies. The method of outcome assessment was thought to be comparable across intervention groups and the outcome measure was considered minimally influenced by the knowledge of intervention. Selective reporting of subgroup of participants (e.g. patients receiving secondary surgery) was observed in one study, evaluated as a serious reporting bias [22].

#### Before-after studies

We adapted the ROBINS-I for evaluating the risk of bias in before-after studies, results are reported in S3 Table and S4 Fig.

All studies, even the MIGS arms RCT and NRS were considered as before-after studies. For before-after studies confounding domain was not applicable as subjects were not assigned to different groups. Seven out of 30 studies [24,25,30,33,48,50,51] recruited consecutive patients, thus they were at low risk of bias in selection of participants; nine papers [22,23,26,27,28,34,38,42,47] were judged at serious risk of selection bias because the exclusion of some eligible participants was related to the outcome (e.g. exclusion of patients who did not complete the follow-up or received secondary surgery). In some studies lack of information about selection of participants could not permit risk assessment. Misclassification of assignment of intervention did not occur in before-after study as for NRS: MIGS and cataract surgery are well-defined once-only interventions.

Glaucoma therapy was the main cointervention, potentially causing serious performance biases. Only studies which reported washed-out IOP both pre- and post-operatively were judged to be at low risk [31,36,37,43,46]; other studies were considered at moderate risk if one between pre and post-operative IOP measure was washed-out. Only one study reported masking strategy [43]. Risk of bias due to missing data, measurement of outcome and selection of the reported results were similar to those of NRS.

Funding and authors affiliations for all studies are reported in S4 Table.

### Outcomes in comparative studies

**Fig 2:**
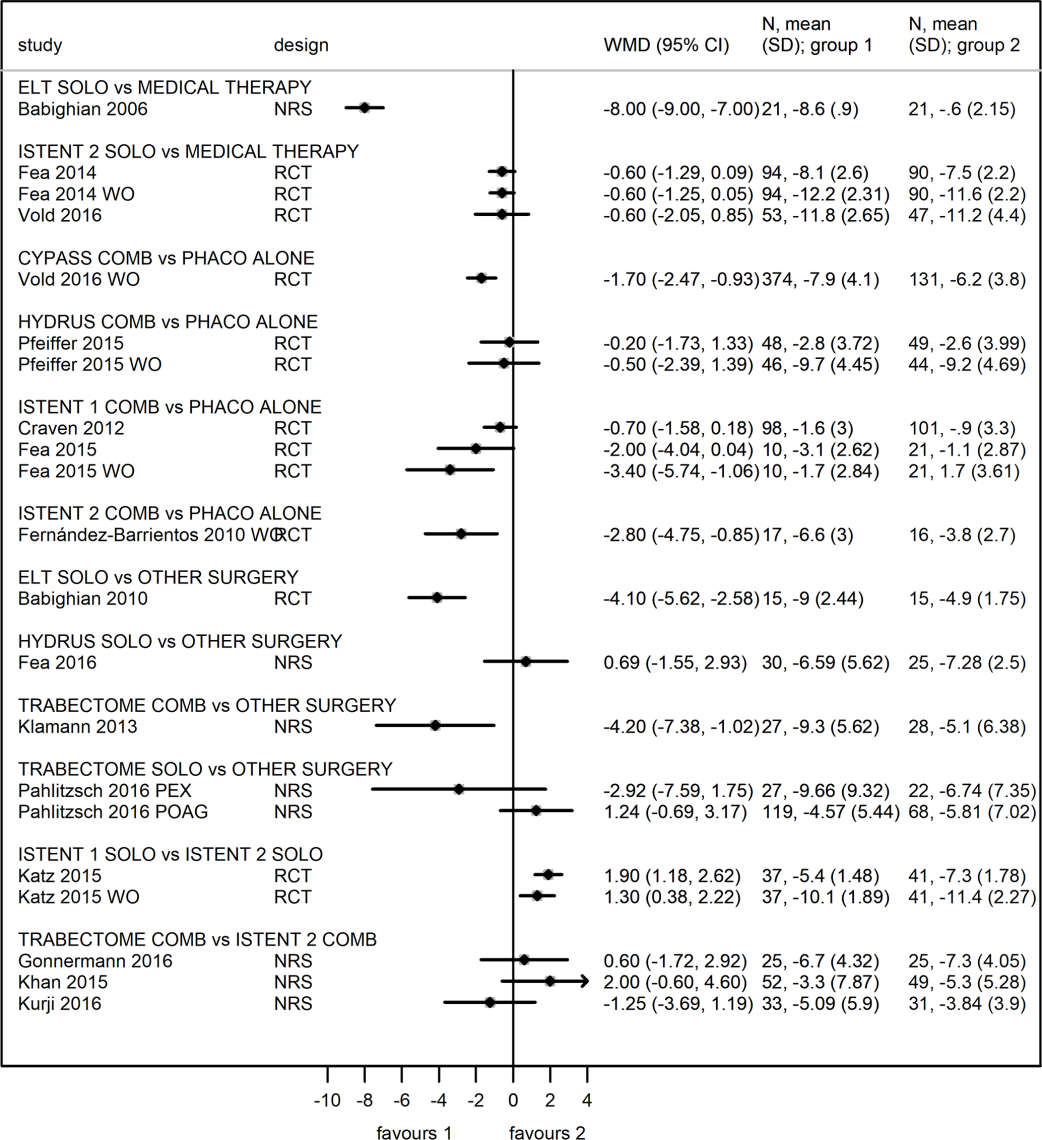
Forest plot for comparison in IOP change between study arms at 12-months (divided by device and procedure). Values expressed in Weighed Mean Difference (WMD).

**Fig 3:**
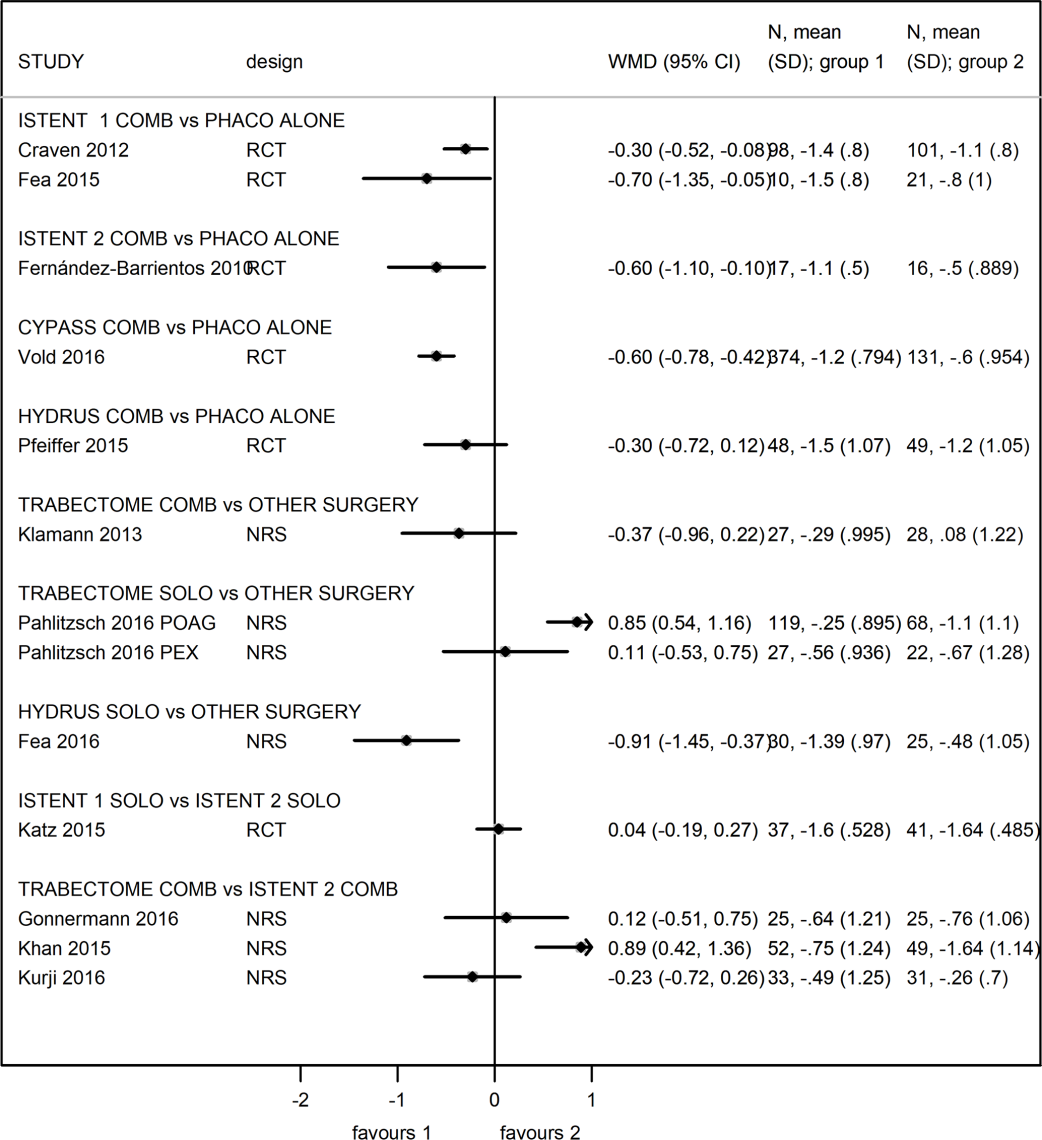
Forest plot for 12-months difference in change in number of glaucoma medications reduction (divided by device and procedure) values expressed in Weighed Mean Difference (WMD).

Outcomes are reported in Figs 2 and 3.

#### MIGS devices versus cataract surgery

Five papers (all RCT) compared MIGS and cataract surgery with cataract surgery alone on a total of 871 patients. CYPASS: one RCT [43] compared combined Cypass surgery with cataract surgery alone on 505 patients. Unmedicated IOP data were reported and showed at 1 year a mean change of 7.9 ± 4.1 mmHG and 6.2 ± 3.8 mmHg in the MIGS and control groups respectively. WMD was -1.7 (95% CI -2.47, -0.93) in favor of Cypass surgery (Fig 2). The change in the number of glaucoma medication was 1.2 ± 0.79 and 0.6 ± 0.95 in the MIGS and control group respectively. WMD was -0.6 (95% CI -0.78, -0.42) in favor of Cypass surgery (Fig 3). The IOP lowering effect of the Cypass remained stable over 2 years, as well as the glaucoma medication reduction (S5 and S6 Figs). Risk of bias analysis showed unclear risk in four domains and low risk in two domains (S1 Table).

HYDRUS: one RCT [46] compared combined Hydrus surgery with cataract surgery alone on 100 patients. Medicated IOP data were reported and showed at 1 year a mean change of 2.8 ± 3.7 mmHg and 2.6 ± 4 mmHg in the MIGS and control groups respectively. WMD was -0.2 (95% CI -1.73, 1.33) in favor of Hydrus surgery (Fig 2). Unmedicated IOP data were reported and showed at 1 year a mean change of 9.7 ± 4.5 mmHg and 9.2 ± 4.7 mmHg in the MIGS and control groups respectively. WMD was -0.5 (95% CI -2.39, 1.39) in favor of Hydrus surgery. The change in the number of glaucoma medication was 1.5 ± 1.07 and 1.2 ± 1.05 in the MIGS and control group respectively (Fig 3). WMD was -0.3 (95% CI -0.72, 0.12) in favor of Hydrus surgery. The IOP lowering effect of the Hydrus remained stable over 2 years, as well as the glaucoma medication reduction (S5 and S6 Figs). These results suggest little difference in IOP change between Hydrus surgery and cataract surgery. The study showed low risk of bias in five domains (S1 Table).

ISTENT: three RCT [30,31,35] compared combined iStent surgery with cataract surgery alone on 266 patients.

In the RCT by Craven [30], medicated IOP data were reported and showed at 1 year a mean change of 1.6 ± 3 mmHg and 0.9 ± 3.3 mmHg in the MIGS and control groups respectively. WMD was -0.7 (95% CI -1.58, 0.18) in favor of iStent surgery (Fig 2). The change in the number of glaucoma medication was 1.4 ± 0.8 and 1.1 ± 0.8 in the MIGS and control group respectively. WMD was -0.3 (95% CI -0.52, -0.08) in favor of iStent surgery (Fig 3). The IOP lowering effect of the iStent remained stable over 2 years, as well as the glaucoma medication reduction (S5 and S6 Figs). Risk of bias analysis showed unclear risk in four domains and low risk in two domains (S1 Table).

In the RCT by Fea [31], medicated IOP data were reported and showed at 1 year a mean change of 3.1 ± 2.6 mmHg and 1.1 ± 2.9 mmHg in the MIGS and control groups respectively. WMD was -2.0 (95% CI -4.04, 0.04) in favor of iStent surgery. Unmedicated IOP data were reported and showed at 1 year a mean change of—1.7 ± 2.8 mmHg and 1.7 ± 3.6 mmHg in the MIGS and control groups respectively. WMD was -3.4 (95% CI -5.74, -1.06) in favor of iStent surgery (Fig 2). The change in the number of glaucoma medication was 1.5 ± 0.8 and 0.8 ± 1 in the MIGS and control group respectively. WMD was -0.7 (95% CI -1.35, -0.05) in favor of iStent surgery (Fig 3). This study was at high risk of attrition bias and low or unclear risk of other biases (S1 Table).

In the RCT by Fernández-Barrientos [35], IOP data were reported as unmedicated and medicated values at baseline and 1 year after double iStent surgery respectively. The mean change in IOP was 6.6 ± 3 mmHg and 3.8 ± 2.7 mmHg in the MIGS and control groups respectively. WMD was -2.8 (95% CI -4.75, -0.85) in favor of iStent surgery (Fig 2). Risk of bias analysis showed unclear risk in four domains and low risk in two domains (S1 Table).

Meta-analysis was performed considering the studies by Craven [30] and Fea [31], with moderate heterogeneity (I^2^ = 24.3%). WMD was -1.01 (95% CI -2.1, 0.08); p = 0.07 suggesting a small advantage of iStent compared to cataract surgery, but this estimate was imprecise and the 95%CI included no difference.

#### MIGS devices versus medical therapy

Three studies (2 RCTs and 1 NRS, 335 patients overall) compared MIGS (ELT and iStent as solo procedures) with standard medical therapy.

ELT: in the NRS by Babighian [50], ELT was compared to medical therapy that was carried-on in fellow eyes, representing the control group. This study with a small sample size (21 patients) showed a large statistically significant IOP difference, WMD = -8.0 mmHg (95% CI -9.0, -7.0); IOP change was -8.6 ± 0.9 mmHg and -0.6 ± 2.2 mmHg in the ELT and medication group, respectively (Fig 2). The study reported outcomes on the number of glaucoma medications at 2 years only, with a WMD of -1.53 (95% CI -1.96, -1.10) in the ELT group compared to baseline values (S6 Fig). IOP changes at 2 years were similar to those at 12 months (S5 Fig). This study was at high risk of bias in two domains of the tools (bias due to confounding, bias due to deviation from intended intervention), S2 Table.

ISTENT: in the RCT by Fea [39] (94 eyes in the iStent group and 98 eyes in the medication group) the medicated IOP change was 8.1 ± 2.6 mmHg in the 94 eyes with double iStent implant and 7.5 ± 2.2 mmHg in the 98 eyes on medical therapy (beta-blocker and prostaglandin). The WMD was -0.60 (95% CI -1.29, 0.09) in favour to iStent surgery. Considering baseline wash-out IOP values, IOP changes showed greater reduction in both groups: 12.2 ± 2.3 and 11.6 ± 2.2 in iStent and medication group respectively. The WMD did not show any difference, -0.60 (95% CI -1.25, 0.05) in favour to iStent surgery (Fig 2). Change analysis in number of medications could not be performed because all patients were on one anti-glaucomatous drug before randomization. Risk of bias analysis showed unclear risk in four domains and low risk in two domains (S1 Table).

In the RCT by Vold [41] 54 naïve eyes were randomized to double iStent implantation and 47 eyes to medical therapy (prostaglandin). It emerged a greater IOP reduction in the iStent group (11.8 ± 2.65 mmHg) than in the medication group (11.2 ± 4.4 mmHg), WMD = -0.60 (95%CI -2.05, 0.85), Fig 2. Analysis on medication change was not conducted because of per-protocol use of anti-glaucomatous medications. The study was at high risk of bias in one domain (S1 Table).

Meta-analysis was performed on these two studies. Implantation of 2 iStent caused a slightly larger reduction in IOP compared to medical therapy, WMD = -0.60 (95% CI -1.23, 0.03), I-squared = 0.0%, p = 0.060; however this benefit was modest and the 95% CI included no difference.

#### MIGS devices versus other glaucoma surgeries

Four papers (1 RCT, 3 NRS including 30 and 347 patients respectively) compared MIGS with other glaucoma surgeries. In three studies (1 RCT and 2 NRS) MIGS were performed as solo procedures while in one NRS MIGS and its comparator were performed together with cataract surgery.

ELT: one RCT [51] compared ELT surgery with SLT on 30 patients. Medicated IOP data were reported and showed at 1 year a mean change of 9.0 ± 2.4 mmHg and 4.9 ± 1.8 mmHg in the MIGS and control groups respectively. WMD was -4.1 (95% CI -5.62, -2.58) in favor of ELT surgery (Fig 2). The change in the number of glaucoma medication was not reported at one year. At two years after surgery the IOP change in the ELT group was slightly inferior than it was after 12 months (S2 Fig). Glaucoma medication were reported at 2 years, with a WMD of -1.54 (95% CI -2.05, -1.03) in the ELT group compared to baseline values. The study was at low risk of bias in all domains but one which was judged unclear (S1 Table).

HYDRUS: one NRS [48] compared combined Hydrus surgery with SLT on 56 patients. Medicated IOP data were reported and showed at 1 year a mean change of 6.6 ± 5.6 mmHg and 7.3 ± 2.5 mmHg in the MIGS and SLT group respectively. WMD was 0.69 (95% CI -1.55, 2.93) in favor of SLT (Fig 2). The change in the number of glaucoma medication was 1.39 ± 0.97 and 0.48 ± 1.1 in the MIGS and SLT group respectively. WMD was -0.91 (95% CI -1.45, -0.37) in favor of Hydrus surgery (Fig 3). The study was at moderate risk of bias in three domains and at low risk in four domains (S3 Table).

TRABECTOME: one study (NRS) compared Trabectome and cataract surgery with trabecular aspiration and cataract surgery on 55 patients [24]. In the study, medicated IOP data were reported and showed at 1 year a mean change of 9.3 ± 5.6 mmHg and 5.1 ± 6.4 mmHg in the MIGS and control groups respectively. WMD was -4.2 (95% CI -7.38, -1.02) in favor of Trabectome combined surgery (Fig 2). The change in the number of glaucoma medication was 0.29 ± 1.0 and -0.08 ± 1.22 in the MIGS and control group respectively. WMD was -0.37 (95% CI -0.96, 0.22) in favor of Trabectome surgery (Fig 3). The study was at serious risk of bias in one domain and at moderate and low risk in other domains (S2 Table).

One study (NRS) compared Trabectome surgery with a modified goniotomy technique on 49 PEX and 187 POAG patients [28]. Medicated IOP data on PEX patients were reported and showed at 1 year a mean change of 9.7 ± 9.3 mmHg and 6.7 ± 7.4 mmHg in the MIGS and control groups respectively. WMD was -2.92 (95% CI -7.59, 1.75) in favor of Trabectome surgery. Different results were reported in POAG patients where mean IOP change at 1 year was 4.6 ± 5.4 mmHg and 5.8 ± 7.0 mmHg in the MIGS and control groups respectively. WMD was 1.24 (95% CI -0.69, 3.17) in favor of modified goniotomy surgery (Fig 2). The change in the number of glaucoma medication in the PEX cohort was 0.56 ± 0.94 and 0.67 ± 1.28 in the MIGS and control group respectively. WMD was 0.11 (95% CI -0.53, 0.75) in favor of modified goniotomy surgery. Similar findings were observed in the POAG cohort where the change in the number of glaucoma medication was 0.25 ± 0.9 and 1.1 ± 1.1 in the MIGS and control group respectively. WMD was 0.85 (95% CI 0.54, 1.16) in favor of modified goniotomy surgery (Fig 3). The study was at serious risk of bias in two domains (S2 Table).

#### MIGS procedures versus other MIGS procedures

1 ISTENT versus 2 and 3 ISTENT: in the RCT by Katz, 38 subjects were implanted with one stent, 41 subjects with two stents, and 40 subjects with three stents. At 12 months, a greater efficacy of 2 versus 1 iStent implantation has been demonstrated, with mean differences in reduction of 1.90 mmHg (95% CI 1.18–2.62). Higher IOP reduction resulted after 3 iStent implantation, with mean IOP change from baseline of 8.2 mmHg compared to 5.4 mmHg after 1 iStent implantation (only results on 1 and 2 iStent are showed in Fig 2). Considering wash-out IOP, the difference in reduction between 1 and 2 iStent was 1.30 mmHg (95% CI 0.38, 2.22), in favor to 2 iStent. Number of medication decreased of 1.60 drug/patient in the 1iStent group, 1.64 in the 2 iStent group and 1.43 in the 3 iStent group. This study showed a high risk of performance and detection bias because of lack of masking (S1 Table).

TRABECTOME versus ISTENT: three studies compared Trabectome to iStent surgery combined [22, 23, 25]. In these three NRS, 2 iStent were implanted in a total of 108 patients with POAG and cataract, while Trabectome and phacoemulsification were performed in 113 patients. In the NRS by Gonnermann [22], slightly greater IOP reduction was observed in the iStent arm (25 eyes), with IOP change of 7.3 ± 4.1 mmHg, compared to 6.7 ± 4.3 mmHg in the Trabectome arm (25 eyes), WMD = 0.60 (95% CI -1.72, 2.92), Fig 2. Results of number of medication showed greater reduction in the iStent group (0.76 ± 1.06 drug/patient) compared to the Trabectome group (0.64 ± 1.21), but this was not statistically significant (WMD = 0.12, 95% CI -0.51, 0.75), Fig 3. This study was at serious risk of bias in four domains (S2 Table).

Khan [23] reported that iStent group (49 eyes) achieved greater IOP reduction in comparison to the Trabectome group of 52 eyes (5.3 ± 5.3 mmHg vs 3.3 ± 7.9 mmHg respectively) resulting in 2 mmHg of difference (95% CI -0.60, 4.60), Fig 2. Difference in number of medication was in favor to iStent procedure, WMD = 0.89 (CI 0.42, 1.36), Fig 3. This study was at serious risk of bias in three domains (S2 Table).

Kurji [25] reported greater effect in the Trabectome (36 eyes) than in the iStent (34 eyes) arm: IOP change was respectively 5.1 ± 5.9 mmHg and 3.8 ± 3.9 mmHg, WMD = -1.25 (95% CI -3.7, 1.2), Fig 2. Number of medication change was respectively 0.49 ± 1.25 drug/patient and 0.26 ± 0.7 drug/patient, WMD = -0.23 (95% CI -0.72, 0.26), Fig 3. This study was at serious risk of bias in two domains (S2 Table).

The meta-analysis of these three studies on IOP change, with moderate heterogeneity (I-square = 24.3%), showed no difference between Trabectome and iStent as combined procedures, WMD = 0.41 (95% CI -1.40, 2.21), p = 0.65. Meta-analysis on the number of medication was not carried out due to high heterogeneity (I-squared = 73.2%).

### Outcomes in before-after studies

#### Iop change

**Fig 4:**
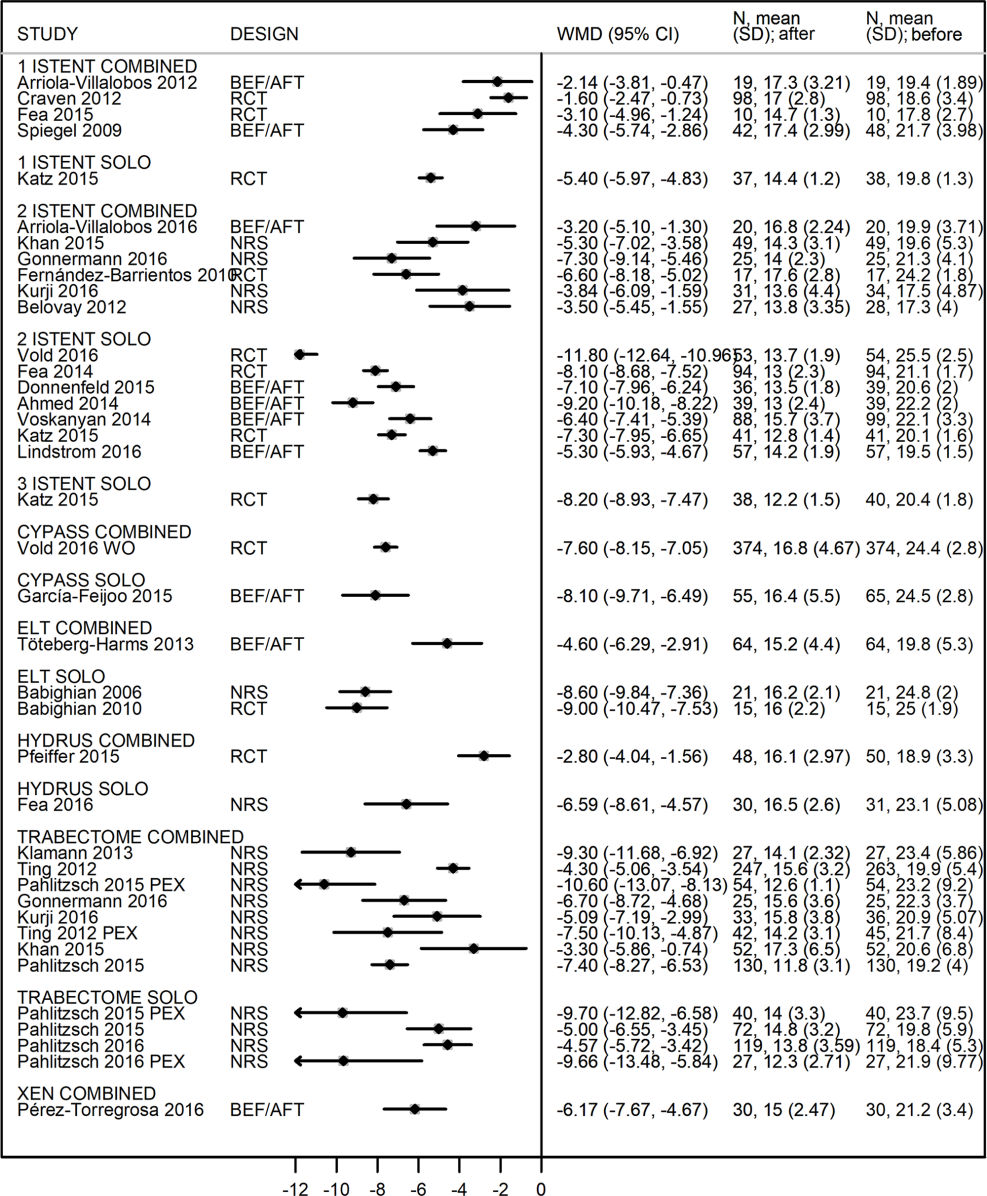
Forest plot for 12-months IOP reduction (divided by device and procedure).Values expressed in Weighed Mean Difference (WMD).

Considering all papers as before-after studies (only MIGS arm in the RCT and NRS), all the MIGS procedures result in a significant reduction of IOP (Fig 4).

The highest reductions was achieved by the study considering naïve patients (WMD = -11.8 in Vold study [41]) followed by studies on Trabectome in PEX subgroups (WMD = -10.60 [26], -9.70 and -9.66 [26, 28]). ELT confirmed the hypotensive efficacy showed in the comparative analysis (WMR = -9 [51] and -8.60 [50] in the RCT and NRS respectively). As regards the iStent, combined procedures showed a lower IOP reduction compared to solo procedure.

The Hydrus combined study [46] showed a WMD of -2.80 mmHg at one year after surgery, lower than that observed in the Hydrus as a solo procedure study (WMD = -6.59) [48].

Two years after surgery, overall IOP reduction was similar to that found at one year: WMD ranged from -1.50 [30] to -11.70 in a wash-out study [43]., although a smaller number of studies was considered (S1 Fig).

#### Number of glaucoma medications

Five studies did not report outcomes on the number of glaucoma medications at one year [38,42,47,50,51].

Four studies reported the number of medications used per protocol [37–40] while one study reported none glaucoma medication at last follow-up [35].

Finally, one study was conducted on glaucoma naive patients [41].

**Fig 5:**
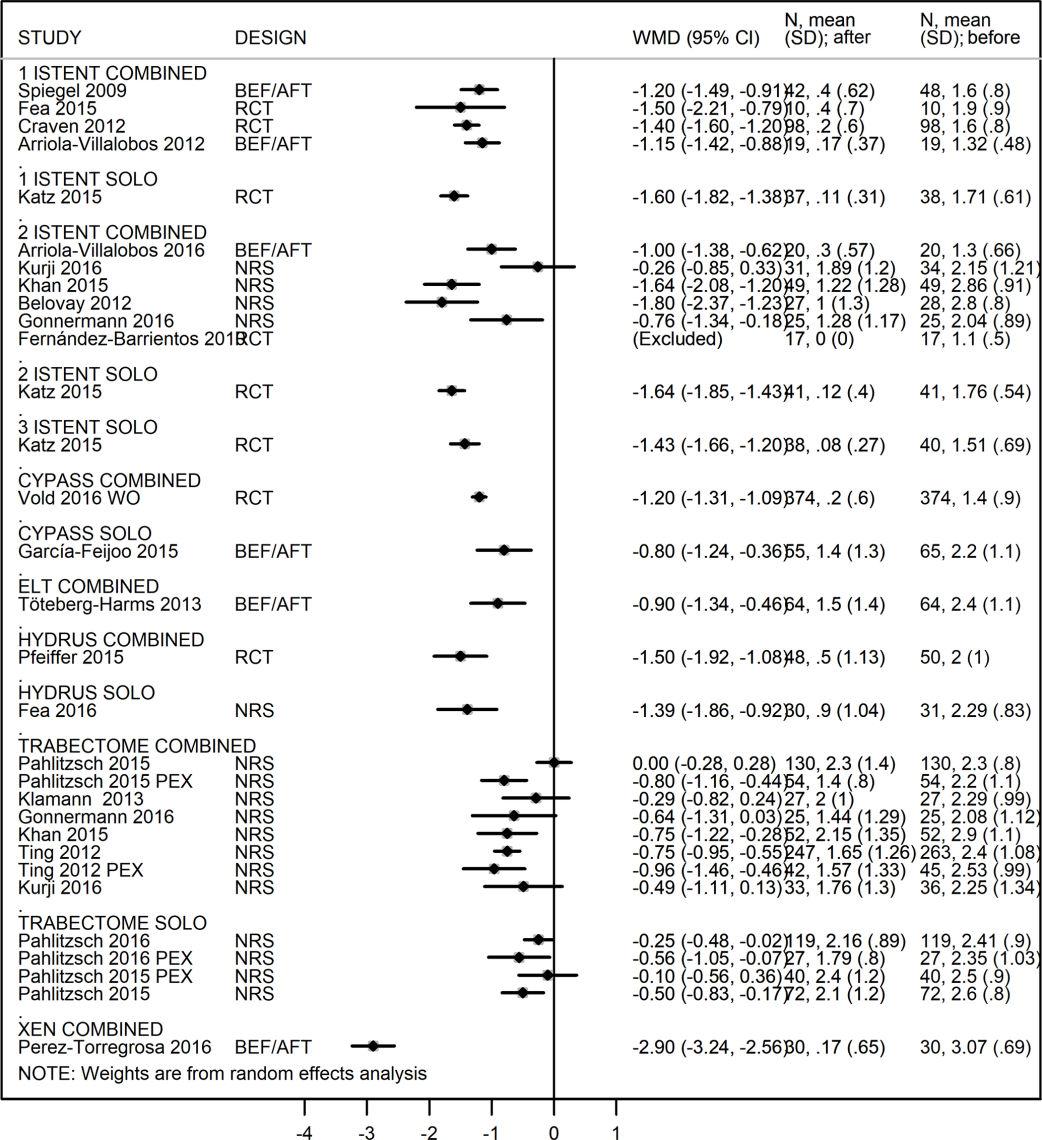
Forest plot for 12-months number of medication reduction (divided by device and procedure). Values expressed in Weighed Mean Difference (WMD).

Thus, Forest plot of the different in change in glaucoma medication reduction comprised a smaller number of studies (Fig 5).

The XEN [45] as a combined procedure and the iStent [36] as a solo procedure gave the highest reduction in glaucoma medications compared to before surgery values (WMD = -2.90 and WMD = -1.60 drugs/patient respectively).

Two years after surgery, glaucoma medication reduction was apparently higher compared to 1-year: all WMD, except one, were over 1 drug/patient (S2 Fig). However, the number of papers available for 2 year-analysis was low (n = 8 [29,30,33,43,46,47,50,51]) and studies with both one and two-year data did not show glaucoma medication reduction between these timelines [29,30,33,43,46]. Three studies [47,50,51] provided a medication reduction report at two years only.

### Adverse outcomes

Postoperative ocular adverse events are summarized in Table 5.

**Table 5:** Surgery-related adverse events of included studies.

Device	Author, Year	Eye (N)	FOLLOW-UP (MONTHS)	IOP SPIKE N (%)	INFECTION N (%)	HYPOTONY N (%)	BCVA LOSS >2 LINES DUE TO GLAUCOMA N (%)	ADDITIONAL DEVICE-RELATED PROCEDURE N (%)	ADDITIONAL SURGERY N (%)
**TRABECTOME**									
COMBO									
	GONNERMANN, 2016^22^	27	12	NR	0	0	0	NR	2/27 (7.4)
	KHAN, 2015^23^	52	12	17/52 (32.7)	0	0	NR	3/52 (5.8)^a^	4/52 (7.7)
	KURJI, 2016^25^	36	12	2/36 (5.6)	NR	NR	NR	NR	3/36 (8.3)
	PAHLITZSCH, 2015 (POAG)^26^	130	12	*	*	*	*	*	*
	PAHLITZSCH, 2015 (PEX)^26^	54	12	*	*	*	*	*	*
	TING, 2012 (POAG)^27^	263	12	26/263 (9.9)	0	0	0	NR	16/263 (6.1)
	TING, 2012 (PEX) ^27^	45	12	4/45 (8.9)	0	1/45 (2.2)	0	NR	3/45 (6.7)
SOLO									
	PAHLITZSCH, 2015 (POAG)^26^	72	12	*	*	*	*	*	*
	PAHLITZSCH, 2015 (PEX)^26^	40	12	*	*	*	*	*	*
	PAHLITZSCH, 2016 (POAG)^28^	119	12	0	0	0	0	NR	5/119 (4.2)
	PAHLITZSCH, 2016 (PEX)^28^	27	12	0	0	0	0	NR	0
**ISTENT**									
COMBO									
1 STENT									
	ARRIOLA-VILLALOBOS, 2012^29^	19	60	4/19 (21)	*	0	0	0^b^	0
	CRAVEN^c^, 2012^30^	116	24	5/116 (4.3)	0	0	0	5/116 (4.3)	1/116 (0.9)
	FEA, 2015^31^	10	48	0^b^	0^b^	0^b^	0^b^	0^b^	0
	SPIEGEL^c^, 2009^32^	58	12	1/58 (1.7)^d^	NR	0	0	7/58 (12)^e^	2/58 (3.5)
2 STENT									
	ARRIOLA-VILLALOBOS 2016 (INJECT)^33^	20	12	3/20 (15)	0	0	0	1/20 (5)	0
	BELOVAY^f^, 2012^34^	28^f^	12	*	*	*	0/53	6/53 (11.3) ^f^	*
	GONNERMANN, 2016 (INJECT)^22^	27	12	NR	0	0	0	NR	2/27 (7.4)
	KHAN, 2015^23^	49	12	8/49 (16.3)	0	2/49 (4.1)	NR	3/49 (6)^d^	0
	KURJI, 2016^25^	34	12	2/34 (5.9)	NR	NR	NR	NR	0
SOLO									
1 STENT									
	KATZ, 2015^36^	38	18	*	*	*	*	*	0
2 STENT									
	AHMED, 2014^37^	39	18	*	*	1/39 (2.6)	0	*	*
	DONNENFELD, 2015^38^	39	36	1/39 (2.6)	*	0	0	1/39 (2.6)^g^	*
	KATZ, 2015^36^	41	18	*	*	*	*	*	0
	FEA, 2014 (INJECT)^39^	94	12	1/94 (1.1)	0^b^	0^b^	0^b^	1/94 (1.1)	0^b^
	LINDSTROM, 2016 (INJECT)^40^	57	18	*	*	*	*	*	0
	VOLD, 2016^41^	54	36	*	*	*	*	*	*
	VOSKANYAN, 2014 (INJECT)^42^	99	12	10/99 (10.1)	0	0	0	4/99 (4)	4/99 (4)
3 STENT									
	KATZ, 2015 (ISTENT)^36^	40	18	*	*	*	*	*	0
**CYPASS**									
COMBINED									
	VOLD, 2016^43^	374	25	16/374 (4.3)	0	11/374 (2.9)	4/374 (1.1)^h^	NR^i^	3/374 (0,8)
SOLO									
	GARCÍA-FEIJOO, 2015^44^	65	12	7/65 (10.8)	0	0	0	1/65 (1.5)^j^	12/65 (18.5)
**XEN**									
COMBO									
	PEREZ-TORREGROSA, 2016^45^	30	12	*	*	0	0	7/30 (23.3)	*
**HYDRUS**									
COMBO									
	PFEIFFER, 2015^46^	50	24	2/50 (4)	0	0	0	NR	1/50 (2.1)
SOLO									
	GANDOLFI, 2016^47^	21	24	1/21 (4.76)	NR	NR	NR	4/21 (19.05)	2/ 21 (9.5)
	FEA, 2016^48^	31	12	2 (6.5)	0^b^	0^b^	0^b^	0^b^	0^b^
**ELT**									
COMBO									
	TÖTEBERG-HARMS, 2013^49^	64	12	NR	0	NR	NR	NR	7/64 (10.9)
SOLO									
	BABIGHIAN, 2006^50^	21	24	*	*	0	*	*	*
	BABIGHIAN, 2010^51^	15	24	3/15 (20)	0	NR	NR	NR	NR

SD = standard deviation. POAG = primary open angle glaucoma. Pex = pseudoexfoliative glaucoma. IOP = intraocular pressure. BCVA = best corrected visual acuity. NR = not reported; G1 = iStent. G2 = iStent inject

* = reported in the manuscript as “no serious postoperative complications”.

^a^ = 2 goniopuncture, 1 revision

^b^ = reported by corresponding author.

^c^ = safety analysis performed on intent-to treat population.

^d^ = reported as "corneal paracentesis to reduce IOP".

^e^ = 2 replacement,1 reposition, 1 argon-laser to remove iris tissue in contact with iStent lumen, 2 TPA, 1 corneal paracentesis.

^f^ = safety reported for 2-stent and 3-stent group together. YAG or argon laser for stent blockage.

^g^ = surgical irrigation of the anterior chamber for hyphema.

^h^ = not specify if related to POAG

^i^ = 19 secondary surgery, not specified.

^j^ = YAG-laser synechiolyis.

Adverse events were reported in most studies while some others just reported sentences like “no serious adverse event were observed”.

There was no report of postoperative infection or BCVA loss > 2 lines due to glaucoma. The most frequent adverse events were IOP spikes, that occurred in the iStent, in the Cypass and in the Hydrus both as solo (iStent range 1.1% - 10.1% [39,42]; Cypass 10.8% [44]; Hydrus 4.76% and 6.5% [47,48]) and combined procedures (iStent range 0%-21% [31,29]; Cypass 4.3% [43]; Hydrus 4% [46]). In the Trabectome and in the ELT, IOP spikes were respectively reported just for combined (range 5.6%-32.7% [25,23]) or solo procedures (20% [51]).

Additional surgery included trabeculectomy, shunt / valve implant, cyclophotocoagulation, deep sclerectomy or other MIGS procedures.

## Discussion

We have performed a systematic review of the studies comparing MIGS techniques, alone or combined with cataract surgery, with medical or laser therapy, cataract surgery or other MIGS techniques.

This paper presents results from studies with three different designs: RCT, NRS and before-after. One-year results of RCT and NRS have been presented together while before-after studies have been reported separately with MIGS arms from both RCT and NRS. In general, due to the higher intrinsic value of RCT, it’s difficult to compare their results with those from other study subtypes. However, due to the high number of non RCT papers and the growing interest on MIGS, it seemed reasonable to include even NRS and before-after. To avoid as much as possible biases deriving from low quality series, we applied strict inclusion-exclusion criteria to NRS and before-after (e.g. patients lost to follow-up > 15%, previous glaucoma surgery). Moreover, meticulous risk of bias assessment has been conducted on all studies, trying to underline their limits and to inform the scientific community about the necessity of well-structured, independent RCTs. The reader should be cautious when interpreting the results we have reported.

Due to the recent introduction of MIGS, we decided to confine our analysis to one-year follow-up data. Nevertheless, two-years analysis of IOP and glaucoma medications, when available, has been provided in the supplementary matherial. Considering that glaucoma is a chronic disease, two-year data are somehow limited and longer follow-up data is awaited and will certainly be available as soon as MIGS become more widely used. The potential effect of MIGS on subsequent filtering surgery is still to be investigated and any opinion on this matter can only be speculative.

### Efficacy analysis

#### RCTs

Only nine RCTs were found [30,31,35,36,39,41,43,46,51], three with small sample sizes (< 100 patients) [31,35,51] on four different MIGS devices (i.e. iStent, Cypass, Hydrus and ELT).

Five RCTs [30,31,35,43,46] compared MIGS and cataract surgery to cataract surgery alone, and one study with a small sample size compared ELT with SLT [51], with the MIGS showing higher IOP and glaucoma medication reduction.

The design of the studies comparing iStent with medical therapy [39,41] was different: in one study patients with uncontrolled glaucoma on one medication were randomized to either a combined drug (beta-blocker and prostaglandin) or to the implant of two iStents [39]; the other study was done in naïve patients randomized to either two iStent or to a prostaglandin [41]. The implantation of the two iStents obtained a larger reduction of IOP than the comparator group in both studies. Of particular interest is the study on naïve patients in which the comparator (one prostaglandin) presented a mean reduction in IOP of 11.2 mm Hg corresponding to a 44.6% IOP reduction, somehow more than the 25–35% generally reported in the literature [54].

One study compared the efficacy of one vs multiple (two or three) iStents implanted in patients with uncontrolled IOP on two pre-operative medications [36]. Multiple iStents seem to provide a significant advantage over a single iStent implant.

To avoid the confounding effect of considering at the same time the IOP reduction and the reduction of medical therapy in three studies comparing cataract surgery with combo surgery (MIGS and cataract), the patients were washed-out both at baseline and after surgery [31,43,46]. The iStent [31] and the Cypass [43] studies demonstrated a significant advantage of the combined surgery over cataract alone, while the Hydrus study [46] demonstrated a modest difference compared to cataract surgery alone. This interpretation of the results is somehow misleading if the absolute IOP change of the MIGS group and the comparator are not taken into account. The absolute IOP reduction in the washed-out patients was higher in the Hydrus study (9.7±4.45 mm Hg) compared to the Cypass (7.9±4.1 mm Hg) and the iStent (1.7± 2.84 mmHg) studies. In the Hydrus study the difference with the comparator was relatively low due to a greater IOP reduction observed in the comparator group (cataract: 9.2±4.69 mm Hg). Some of the potential inconsistencies regarding the efficacy of the MIGS procedures when compared to cataract surgery can thus be due to the variability of IOP reduction in the cataract surgery group reported in the different studies. The reduction of IOP following cataract surgery reported elsewhere in the literature is of approximately 5 mmHg [55]. Some of the differences in the IOP reduction after cataract surgery in these studies may be related to the higher baseline IOP as IOP reduction after cataract surgery has been demonstrated to be proportional to the pre-operative IOP.

The IOP lowering effect of MIGS was greater than that of comparators in all studies, although statistical significant difference was borderline or not achieved when meta-analysis was performed. Higher differences were observed in the small ELT vs SLT study by Babighian (4.1 mmHg) [51] and iStent and cataract surgery vs cataract surgery alone by Fea (3.4 mmHg, after 12 months washout) [31] and Fernández-Barrientos (2.8 mmHg) [35]. In RCTs with bigger samples the differences between MIGS and their comparators were smaller, between 0.2 [46] and 1.7 mmHg [43]. However, these data should be evaluated considering the coexisting reduction in glaucoma medication, which was 1.2 [46] and 1.64 [43], thus potentially enlarging the absolute gap between the MIGS and the control arm.

Unfortunately, estimates on the additive IOP lowering effect of medication are impossible to perform, due to the different effect on IOP of different molecules and to the inter-subject variability of response to the medications. Baseline and postoperative wash-out IOP values are therefore highly recommended.

As a matter of fact it is somehow difficult to provide a systematic comparison between the different MIGS devices mainly because of the small number of studies reporting both the pre and post-operative wash-out pressures, but also because of differences in protocols and inclusion criteria.

#### NRS

Several NRS studies were included in our systematic review: one compared ELT to medical therapy [50], one Hydrus to SLT [48], three iStent to Trabectome [22,23,25], one Trabectome to trabecular aspiration [24] and one Trabectome to a modified goniotomy technique [28]. In the ELT, the Hydrus and the Trabectome vs goniotomy studies, the MIGS procedure were not combined with cataract surgery.

ELT proved to be superior to medical therapy and the Trabectome to trabecular aspiration both in terms of IOP and medication reduction, whereas the study comparing Hydrus to SLT demonstrated a similar IOP reduction but a significant advantage in the number of medications after the implantation of the Hydrus device.

A non significant advantage of the Trabectome over a modified goniotomy technique was demonstrated in PEX patients only [28]. The comparison of the Trabectome vs 2 iStent proved either in favour of one method or the other [22,25] but the difference was significant in one study only, showing a greater reduction of IOP and medications in the iStent group [23].

Our meta-analysis did show an acceptable heterogeneity for the iStent studies (iStent combined vs phacoemulsification; iStent solo vs medical therapy and iStent vs Trabectome combined), whereas were poor in the Trabectome vs modified goniotomy study. There were no significant differences between the results obtained in the different groups.

#### Before-after studies

Because of the limited research available on the effect of MIGS, we also reported on the IOP lowering effect in before/after studies and in the MIGS arm of RCTs and NRSs, consistently showing an IOP reduction from 1.60 [30] to 11.8 mmHg [41] and a medication reduction by 0 [26] to 2.9 [45]. Although such information should be interpreted with caution, since the starting IOP control and measurement setting was not well reported, these series suggest a potential for IOP lowering by most MIGS and encourage the conduction of further comparative research using good-quality methods and comparators that are meaningful alternatives in modern glaucoma practice.

#### Solo versus combined studies

Several studies investigated the efficacy of the MIGS (Trabectome [26–28], iStent [36–42], ELT [50,51], Hydrus [47,48], Cypass [44]) as a solo procedure. All the studies were favorable to MIGS both in term of IOP reduction (-4,57 to -11,80) [28,41] and medications reduction (-0.10 to -1.64) [26,36], thus suggesting that the MIGS can be effective independently from cataract surgery.

The observation that several solo procedures (iStent, Hydrus and ELT) outperformed combined surgery is somehow unexpected because the reduction of IOP achieved by phacoemulsification alone should add to the effect of the glaucoma procedures [56–58]. This difference is particularly evident when considering the iStent because of the larger number of studies and could not be attributed to either the implantation of different iStent models not to the implantation of multiple devices. Most of the solo iStent studies were performed later and a possible explanation of their apparent better performance could lie in improved surgical techniques and more appropriate selection criteria. Some of the later iStent studies have been performed in areas where the access to pharmacological treatment may be difficult and those patients may have been less exposed to potential negative effect of prolonged topical therapy.

Although the potential negative effect of the prolonged use of glaucoma medications on the results of MIGS device has never been hypothesized, this has been demonstrated with other laser [59] and surgical procedures [60].

### Safety

The number of complications is missing in some of the studies [26,41], but it is minimal whenever reported. In particular there were no reports of infection or decrease in Best Corrected Visual Acuity (BCVA). IOP spikes incidence ranged from 0 to 32.7% [28,31 and 23]. Revision of the procedure was relatively low for the iStent (0 to 6.89%) [29,31 and 32] and more common following the XEN implantation (23.3%) [45], which relies on the presence of a conjunctival bleb. Additional surgery was done in less than 10% of patients with the exception of the Cypass (18.5%) [44] and the ELT (10.9%) [49].

### Risk of bias

Considering the RCT studies, some methodological issues deserve discussion. In most of the studies a clear definition of the randomization method is lacking. The masking of most RCT has been judged as unclear, but it should be pointed out that masking a surgical procedure can be almost impossible both to the patient and to the examiner due to the fact that the device can sometimes be clearly visible. IOP should ideally be recorded by a masked technician or with a two-person system.

Because of the limited amount of evidence, we expanded our analysis by presenting uncontrolled before/after series as well as data from the MIGS arm of NRS and RCTs to yield a better picture of the knowledge in this field.

We adopted ROBINS-I [16], a recently developed methodological tool, to assess the risk of bias in NRS as well as in uncontrolled before/after studies. However, we caution readers regarding the fact that the use of ROBINS-I to assess before/after series is not validated and should be considered experimental unless a specific version is made available by the developers.

When assessing the methodological quality of non-randomized studies, we paid particular attention to specific issues, which apply to both NRS and uncontrolled before/after studies. First, IOP measurement should have been carefully collected, ideally by personnel unaware of the treatment status and not by the treating physician; a minimum of three IOP measurement or a diurnal curve at the before and after point would be useful to limit random error or regression to the mean. Furthermore, in the studies it should be clearly stated if the patients were consecutive, how many patients were excluded during the study and what was the reason for exclusion and how many patients were lost to follow-up.

To minimize the reciprocal effect of IOP reduction and medical therapy reduction, wash-out is advisable and if not deemed possible a staged re-introduction of medical therapy would be advisable. Following predefined rules, it would be clear, then, that a single therapy would correspond to a certain compound and that the addition of a second medication would correspond to another definite compound.

Unfortunately, in most NRS and before/after studies the Authors failed to provide details suggesting that such high standards were met. Conflict of interest is believed to be a potential source of bias in clinical studies, including RCTs. It should be noted that several studies are sponsored and this applies to both RCT, NRS and before-after studies. Detailed funding by device industry or authors affiliations are reported (S4 Table). In general, larger studies need some kind of sponsorship as it happens to all multicentric drug studies. Considering the independent studies, it should be noted that some may have benefitted from the free use of the device, which again is common in the pharmaceutical trials, where the cost of the drug is nevertheless incomparable to the price of this kind of devices.

### Conclusions

A strength of our review was the inclusion of all available evidence on MIGS techniques, comprising RCTs and NRS and assessing their methodological quality.

Our systematic review has found that, although there is increasing interest on safer, standardized and minimally invasive surgeries, the evidence on the efficacy of MIGS compared to other therapies is still limited and is based on few RCTs of acceptable quality and a larger number of NRS and uncontrolled before/after series.

We suggest that future research should be comparative, ideally randomized, including patients and alternative treatments that are relevant to clinical settings.

The results of this meta-analysis show a decrease of IOP and a reduction of glaucoma medications after MIGS surgery with a low complication rate. This could be potentially very relevant for patients and health care providers, allowing a significant number of POAG patients to reduce their glaucoma medication burden. The remarkable heterogeneity of the studies on this topic suggests the need for additional research to understand how to maximize the utility of these new procedures. The potential influence of prolonged glaucoma medication treatment, as well as the effects of higher pre-operative IOP, patient selection and surgical performances should be examined in future investigations.

Similarly, the impact of these mini-invasive procedures on the reported quality of life of patients and their costs, potentially influencing their diffusion, should be investigated.

## Supporting information

S1 Appendix Search strategy for Medline. (DOCX)

S2 Appendix References of studies excluded on the basis of full text. (DOCX)

S1 Table Risk of bias summary for RCTs: Review authors’ judgements about each risk of bias item for each included study. (DOCX)

S2 Table Risk of bias summary for non-RCTs NRS: Review authors’ judgements about each risk of bias item for each included study. (DOCX)

S3 Table Risk of bias summary for non-RCTs Before-after studies: Review authors’ judgements about each risk of bias item for each included study. (DOCX)

S4 Table Risk of bias sponsor-related for all studies. (DOCX)

S1 Fig Risk of bias summary for RCTs: Review authors’ judgements about each risk of bias item for each included study. (DOCX)

S2 Fig Methodological quality graph for RCTs: Review authors’ judgements about each methodological quality item presented as percentages across all included RCTs. (DOCX)

S3 Fig Methodological quality graph for non-RCTs (NRS): Review authors’ judgements about each methodological quality item of ROBINS-I presented as percentages across all included RCTs. (DOCX)

S4 Fig Methodological quality graph for non-RCTs (Before-after study): Review authors’ judgements about each methodological quality item of ROBINS-I presented as percentages across all included RCTs. (DOCX)

S5 Fig Forest plot for 24-months IOP reduction. Values expressed in Weighed Mean Difference (WMD).
(DOCX)

S6 Fig Forest plot for 24-months glaucoma medication reduction. Values expressed in Weighed Mean Difference (WMD).
(DOCX)

S7 Fig PRISMA checklist. (DOCX)
