# Supplementary material for: Minimally-invasive glaucoma surgeries (MIGS) for open angle glaucoma: A systematic review and meta-analysis
Source: PLoS One. 2017 Aug 29;12(8):e0183142. doi: 10.1371/journal.pone.0183142 (PMC5574616; doi:10.1371/journal.pone.0183142)
Supplement: S1 Appendix — (DOCX) [file pone.0183142.s001.docx]

**S1 Appendix. Search strategy for Medline**

# 1 :

("Glaucoma, Open-Angle"[Mesh] OR "Intraocular Pressure"[Mesh] OR "Ocular Hypertension"[Mesh] OR (OAG OR POAG OR IOP OR OHT) OR (simple near/3 glaucoma) OR (open near/2 angle near/2 glaucoma) OR (chronic near/2 glaucoma) OR (low near/2 tension near/2 glaucoma) OR (low near/2 pressure near/2 glaucoma) OR (normal near/2 pressure near/2 glaucoma) OR (normal near/2 tension near/2 glaucoma) OR "Exfoliation Syndrome"[Mesh] OR (exfoliat* near/2 syndrome*) OR (exfoliate* near/2 glaucoma*) OR (pseudoexfoliat* near/2 glaucoma*) OR (pseudoexfoliat* near/2 syndrome*) OR (pigment near/2 glaucoma*))

TRABECTOME

# 1 AND ((ab interno trabeculotomy) OR (trabeculotomy ab interno) OR (trabecular near/2 bypass) OR (ab interno trabeculectomy) OR (trabeculectomy ab interno) OR trabectome)

ISTENT

# 1 AND (iStent OR Glaukos OR (Trabecular Micro Bypass Stent) OR (Trabecular Stent))

HYDRUS

# 1 AND (Hydrus OR Ivantis OR (Schlemm's Canal Scaffold))

CYPASS

# 1 AND (Cypass OR (suprachoroidal Microstent) OR Transcend)

ISTENT SUPRA

#1 AND ((iStent Supra) OR (Glaukos AND Supra) OR (Glaukos iStent Supra) OR (suprachoroidal stent AND (Glaukos OR iStent OR Supra)))

XEN

# 1 AND (Aquesys OR XEN OR (Allergan XEN) OR (Subconjunctival implant) OR (Gel Stent))

AB INTERNO CANALOPLASTY

# 1 AND ((Ab interno canaloplasty) OR (ab near\2 interno) OR (near\2 canaloplasty))

EXCIMER LASER TRABECULOTOMY

# 1 AND ((Excimer Laser Trabeculotomy) OR ELT OR Glautec)

GATT

# 1 AND ((Gonioscopy-assisted transluminal trabeculotomy) OR GATT OR (transluminal trabeculotomy))

FUGO BLADE

# 1 AND ((Singh Filtration) OR (Fugo near\2 blade) OR (transciliary filtration near\ glaucoma))
