# Supplementary material for: Minimally-invasive glaucoma surgeries (MIGS) for open angle glaucoma: A systematic review and meta-analysis
Source: PLoS One. 2017 Aug 29;12(8):e0183142. doi: 10.1371/journal.pone.0183142 (PMC5574616; doi:10.1371/journal.pone.0183142)
Supplement: S2 Appendix — (DOCX) [file pone.0183142.s002.docx]

**S2 Appendix. References of studies excluded on the basis of full text**

**NOTE:** 3 studies (Arriola-Villalobos et al 2013^3^; Fea et al 2010^10^; Samuelson et al 2011^27^) were interim-analysis.

**Inclusion/Exclusion criteria not met.**

TRABECTOME

1. Minckler D, Baerveldt G, Ramirez MA, Mosaed S, Wilson R, Shaarawy T, et al. Clinical results with the Trabectome, a novel surgical device for treatment of open-angle glaucoma. Trans Am Ophthalmol Soc. 2006;104:40-50. **Motivations: glaucoma other than POAG, PEX, PG; previous glaucoma surgery;** **lost at 1-year follow-up > 15%**
2. Francis BA, Minckler D, Dustin L, Kawji S, Yeh J, Sit A, et al.: Trabectome Study Group. et al; Trabectome Study Group. Combined cataract extraction and trabeculotomy by the internal approach for coexisting cataract and open-angle glaucoma: initial results. J Cataract Refract Surg. 2008;34(7):1096-110. **Motivations: glaucoma other than POAG, PEX, PG;** **lost at 1-year follow-up > 15%**
3. Minckler D, Mosaed S, Dustin L, Ms BF; Trabectome Study Group. Trabectome (trabeculectomy-internal approach): additional experience and extended follow-up. Trans Am Ophthalmol Soc. 2008;106:149-159. **Motivations: glaucoma other than POAG, PEX, PG; previous glaucoma surgery;** **lost at 1-year follow-up > 15%**
4. Vold SD, Dustin L; Trabectome Study Group. Impact of laser trabeculoplasty on Trabectome® outcomes. Ophthalmic Surg Lasers Imaging. 2010;41(4):443-451 **Motivations: glaucoma other than POAG, PEX, PG;** **lost at 1-year follow-up > 15%**
5. Ahuja Y, Ma Khin Pyi S, Malihi M, Hodge DO, Sit AJ. Clinical results of ab interno trabeculotomy using the trabectome for open-angle glaucoma: the Mayo Clinic series in Rochester, Minnesota. Am J Ophthalmol. 2013;156(5):927-935.e2. **Motivations: glaucoma other than POAG, PEX, PG; previous glaucoma surgery;** **lost at 1-year follow-up > 15%**
6. Fellman RL, Feuer WJ, Grover DS. Episcleral Venous Fluid Wave Correlates with Trabectome Outcomes: Intraoperative Evaluation of the Trabecular Outflow Pathway. Ophthalmology. 2015;122(12):2385-2391.e1. **Motivations: glaucoma other than POAG, PEX, PG; previous glaucoma surgery;** **lost at 1-year follow-up > 15%**
7. Mizoguchi T, Nishigaki S, Sato T, Wakiyama H, Ogino N. Clinical results of Trabectome surgery for open-angle glaucoma. Clin Ophthalmol. 2015;9:1889-1894. **Motivations: previous glaucoma surgery;** **lost at 1-year follow-up > 15%**
8. Loewen RT, Roy P, Parikh HA, Dang Y, Schuman JS, Loewen NA. Impact of a Glaucoma Severity Index on Results of Trabectome Surgery: Larger Pressure Reduction in More Severe Glaucoma. PLoS One. 2016;11(3):e0151926. **Motivations: glaucoma other than POAG, PEX, PG**
9. Neiweem AE, Bussel II, Schuman JS, Brown EN, Loewen NA. Glaucoma Surgery Calculator: Limited Additive Effect of Phacoemulsification on Intraocular Pressure in Ab Interno Trabeculectomy. PLoS One. 2016;11(4):e0153585. **Motivations: glaucoma other than POAG, PEX, PG**
10. Parikh HA, Bussel II, Schuman JS, Brown EN, Loewen NA. Coarsened Exact Matching of Phaco-Trabectome to Trabectome in Phakic Patients: Lack of Additional Pressure Reduction from Phacoemulsification. PLoS One. 2016;11(2):e0149384. **Motivations: glaucoma other than POAG, PEX, PG**
11. Shoji N, Kasahara M, Iijima A, Takahashi M, Tatsui S, Matsumura K, et al. Short-term evaluation of Trabectome surgery performed on Japanese patients with open-angle glaucoma. Jpn J Ophthalmol. 2016;60(3):156-165. **Motivations: previous glaucoma surgery in POAG group;** **lost at 1-year follow-up > 15% in POAG and PEX groups; no separate data reported (phakic, pseudophakic, combined group)**
12. Akil H, Chopra V, Huang A, Loewen N, Noguchi J, Francis BA. Clinical Results of Ab Interno Trabeculotomy Using the Trabectome in Patients with Pigmentary Glaucoma compared to Primary Open Angle Glaucoma. Clin Experiment Ophthalmol. Published online: 2016 Mar 6. doi: 10.1111/ceo.12737. **Motivations:** **lost at 1-year follow-up > 15%**
13. Yildirim Y, Kar T, Duzgun E, Sagdic SK, Ayata A, Unal MH. Evaluation of the long-term results of trabectome surgery. Int Ophthalmol. Published online: 2016 Feb 9. doi: 10.1007/s10792-016-0190-y. **Motivations: previous glaucoma surgery**
14. Dang Y, Roy P, Bussel II, Loewen RT, Parikh H, Loewen NA. Combined analysis of trabectome and phaco-trabectome outcomes by glaucoma severity. Version 2. F1000Res. 2016 Apr 27 [revised 2016 Jun 27];5:762. doi: 10.12688/f1000research.8448.2. eCollection 2016. **Motivations: glaucoma other than POAG, PEX, PG**
15. Hashemian S J, Miraftabi A, Jafari M E, Hemami M R. Combined cataract extraction and trabeculotomy by the internal approach for coexisting cataract and open-angle glaucoma. Journal of Current Ophthalmology xx (2016) 1-6. In press. **Motivations:** **lost at 1-year follow-up > 15%**

ISTENT

1. Spiegel D, García-Feijoó J, García-Sánchez J, Lamielle H. Coexistent primary open-angle glaucoma and cataract: preliminary analysis of treatment by cataract surgery and the iStent trabecular micro-bypass stent. Adv Ther. 2008;25(5):453-464. **Motivations:** **interim 6-months analysis**
2. Vandewalle E, Zeyen T, Stalmans I. The iStent trabecular micro-bypass stent: a case series. Bull Soc Belge Ophtalmol. 2009;(311):23-29. **Motivations:** **previous glaucoma surgery**
3. Ferguson TJ, Berdahl JP, Schweitzer JA, Sudhagoni RG. Clinical evaluation of a trabecular microbypass stent with phacoemulsification in patients with open-angle glaucoma and cataract. Clin Ophthalmol. 2016 14;10:1767-1773. **Motivations:** **lost at 1-year follow-up > 15%. No exclusion criteria were applied.**
4. Ferguson TJ, Berdahl JP, Schweitzer JA, Sudhagoni R. Evaluation of a Trabecular Micro-Bypass Stent in Pseudophakic Patients With Open-Angle Glaucoma. J Glaucoma. 2016;25(11):896-900. **Motivations:** **lost at 1-year follow-up > 15%. No exclusion criteria were applied.**
5. Gallardo MJ, Supnet RA, Giamporcaro JE, Hornbeak DM. Outcomes of combined trabecular micro-bypass and phacoemulsification in a predominantly Hispanic patient population. Clin Ophthalmol. 2016 11;10:1931-1937. **Motivations:** **previous glaucoma surgery, lost at 1-year follow-up > 15%**
6. **S**eibold LK, Gamett KM, Kennedy JB, Mulvahill MJ, Kroehl ME, SooHoo JR et al. Outcomes after combined phacoemulsification and trabecular microbypass stent implantation in controlled open-angle glaucoma. J Cataract Refract Surg. 2016;42(9):1332-1338. **Motivations: previous glaucoma surgery;** **lost at 1-year follow-up > 15%; glaucoma other than POAG, PEX, PG**
7. Tan SZ, Au L. Manchester iStent study: 3-year results and cost analysis. Eye (Lond). 2016;30(10):1365-1370. **Motivations: glaucoma other than POAG, PEX, PG**

CYPASS

1. Grisanti S, Margolina E, Hoeh H, Rau M, Erb C, Kersten-Gomez I, et al. Supraciliary microstent for open-angle glaucoma: clinical results of a prospective multicenter study. Ophthalmologe. 2014;111(6):548-552 **Motivations: glaucoma other than POAG, PEX, PG**
2. Höh H, Grisanti S, Grisanti S, Rau M, Ianchulev S. Two-year clinical experience with the CyPass micro-stent: safety and surgical outcomes of a novel supraciliary micro-stent. Klin Monbl Augenheilkd. 2014;231(4):377-381. **Motivations: previous glaucoma surgery;** **lost at 1-year follow-up > 15%; glaucoma other than POAG, PEX, PG not specified**
3. Hoeh H, Vold SD, Ahmed IK, Anton A, Rau M, Singh K, et al. Initial Clinical Experience With the CyPass Micro-Stent: Safety and Surgical Outcomes of a Novel Supraciliary Microstent. J Glaucoma. 2016;25(1):106-112. **Motivations: previous glaucoma surgery;** **lost at 1-year follow-up > 15%**

XEN

1. Sheybani A, Lenzhofer M, Hohensinn M, Reitsamer H, Ahmed II. Phacoemulsification combined with a new ab interno gel stent to treat open-angle glaucoma: Pilot study. J Cataract Refract Surg. 2015;41(9):1905-1909. **Motivations: previous glaucoma surgery.**

ELT

1. Pache M, Wilmsmeyer S, Funk J. Laser surgery for glaucoma: excimer-laser trabeculotomy. Klin Monbl Augenheilkd. 2006;223(4):303-307. **Motivations: patients were divided in subgroups on the basis of preoperative IOP. missing data (Number of patients per subgroup)**
2. Wilmsmeyer S, Philippin H, Funk J. Excimer laser trabeculotomy: a new, minimally invasive procedure for patients with glaucoma. Graefes Arch Clin Exp Ophthalmol. 2006;244(6):670-676. **Motivations: lost at 1-year follow-up > 15%; glaucoma other than POAG, PEX, PG.**
3. Töteberg-Harms M, Ciechanowski PP, Hirn C, Funk J. One-year results after combined cataract surgery and excimer laser trabeculotomy for elevated intraocular pressure. Ophthalmologe. 2011;108(8):733-738. **Motivations: glaucoma other than POAG, PEX, PG.**

FUGO BLADE

1. Singh D, Singh K. Transciliary Filtration Using the Fugo Blade. Ann Ophthalmol. 2002; 34 (3):183-187. **Motivations: 6-months analysis**
2. Singh D, Bundela R, Agarwal A, Bist HK, Satsangi SK. Goniotomy ab interno "a glaucoma filtering surgery" using the Fugo Plasma Blade. Ann Ophthalmol (Skokie). 2006;38(3):213-217. **Motivations: 6-months analysis**
3. Dow CT, deVenecia G. Transciliary filtration (Singh filtration) with the Fugo plasma blade. Ann Ophthalmol (Skokie). 2008;40(1):8-14. **Motivations: lost at 1-year follow-up > 15%, follow-up data between 6 and 12 months**

GATT

1. Grover DS, Godfrey DG, Smith O, Feuer WJ, Montes de Oca I, Fellman RL. Gonioscopy-assisted transluminal trabeculotomy, ab interno trabeculotomy: technique report and preliminary results. Ophthalmology. 2014;121(4):855-861. **Motivations: lost at 1-year follow-up > 15%**

**Outcomes of interest not reported**

TRABECTOME

1. Anton A, Neuburger M, Wecker T, Böhringer D, Jordan JF. Body mass index as an influencing factor for outcome of trabectome surgery?. Klin Monbl Augenheilkd. 2014;231(11):1103-1106. **Motivations: no data on IOP/medications**
2. Luebke J, Boehringer D, Neuburger M, Anton A, Wecker T, Cakir B, et al. Refractive and visual outcomes after combined cataract and trabectome surgery: a report on the possible influences of combining cataract and trabectome surgery on refractive and visual outcomes. Graefes Arch Clin Exp Ophthalmol. 2015;253(3):419-423. **Motivations: no data on IOP/medications**
3. Dang Y, Kaplowitz K, Parikh HA, Roy P, Loewen RT, Francis BA, Loewen NA. Steroid-induced glaucoma treated with trabecular ablation in a matched comparison with primary open-angle glaucoma Clin Exp Ophthalmol. 2016 Dec;44(9):783-788. **Motivations: study investigating Trabectome (solo or combined with cataract surgery) in patients with steroid-induced glaucoma (SIG). POAG patients data used for comparison with SIG (general exact matching was applied)**

ISTENT

1. Spiegel D, Wetzel W, Haffner DS, Hill RA. Initial clinical experience with the trabecular micro-bypass stent in patients with glaucoma. Adv Ther. 2007;24(1):161-170 **Motivation: separate data about combined and solo procedure not provided**

CYPASS

1. Saheb H, Ianchulev T, Ahmed II. Optical coherence tomography of the suprachoroid after CyPass Micro-Stent implantation for the treatment of open-angle glaucoma. Br J Ophthalmol. 2014;98(1):19-23**. Motivations:no data on IOP/medications.**

GATT

1. Are the outcomes of circumferential trabeculotomy for adult open angle glaucoma comparable to those of trabeculectomy? **Motivations: lost at 1-year follow-up > 15%.**

**References**

1. Ahmed II, Katz LJ, Chang DF, Donnenfeld ED, Solomon KD, Voskanyan L, et al. Prospective evaluation of microinvasive glaucoma surgery with trabecular microbypass stents and prostaglandin in open-angle glaucoma. J Cataract Refract Surg. 2014;40(8):1295-1300
2. Arriola-Villalobos P, Martínez-de-la-Casa JM, Díaz-Valle D, Fernández-Pérez C, GarcÍa-Sánchez J, García-Feijoó J. Combined iStent trabecular micro-bypass stent implantation and phacoemulsification for coexistent open-angle glaucoma and cataract: a long-term study. Br J Ophthalmol. 2012;96(5):645-649
3. Arriola-Villalobos P, Martínez-de-la-Casa JM, Díaz-Valle D, García-Vidal SE, Fernández-Pérez C, García-Sánchez J, et al. Mid-term evaluation of the new Glaukos iStent with phacoemulsification in coexistent open-angle glaucoma or ocular hypertension and cataract. Br J Ophthalmol. 2013;97(10):1250-1255.
4. Arriola-Villalobos P, Martinez-de-la-Casa JM, Diaz-Valle D, Morales-Fernandez L, Fernandez-Perez C, Garcia-Feijoo J. Glaukos iStent inject® Trabecular Micro-Bypass Implantation Associated with Cataract Surgery in Patients with Coexisting Cataract and Open-Angle Glaucoma or Ocular Hypertension: A Long-Term Study. J Ophthalmol. 2016;2016:1056573.
5. Babighian S, Rapizzi E, Galan A. Efficacy and safety of ab interno excimer laser trabeculotomy in primary open-angle glaucoma: two years of follow-up. Ophthalmologica. 2006;220(5):285-290.
6. Babighian S, Caretti L, Tavolato M, Cian R, Galan A. Excimer laser trabeculotomy vs 180 degrees selective laser trabeculoplasty in primary open-angle glaucoma. A 2-year randomized, controlled trial. Eye (Lond). 2010;24(4):632-638.
7. Belovay GW, Naqi A, Chan BJ, Rateb M, Ahmed II. Using multiple trabecular micro-bypass stents in cataract patients to treat open-angle glaucoma. J Cataract Refract Surg. 2012;38(11):1911-1917.
8. Craven ER, Katz LJ, Wells JM, Giamporcaro JE; iStent Study Group. Cataract surgery with trabecular micro-bypass stent implantation in patients with mild-to-moderate open-angle glaucoma and cataract: two-year follow-up. J Cataract Refract Surg. 2012;38(8):1339-1345
9. Donnenfeld ED, Solomon KD, Voskanyan L, Chang DF, Samuelson TW, Ahmed II, et al. A prospective 3-year follow-up trial of implantation of two trabecular microbypass stents in open-angle glaucoma. Clin Ophthalmol. 2015;9:2057-2065
10. Fea AM. Phacoemulsification versus phacoemulsification with micro-bypass stent implantation in primary open-angle glaucoma: randomized double-masked clinical trial. J Cataract Refract Surg. 2010;36(3):407-412
11. Fea AM, Belda JI, Rękas M, Jünemann A, Chang L, Pablo L, et al. Prospective unmasked randomized evaluation of the iStent inject (®) versus two ocular hypotensive agents in patients with primary open-angle glaucoma. Clin Ophthalmol. 2014;8:875-882
12. Fea AM, Consolandi G, Zola M, Pignata G, Cannizzo P, Lavia C, et al. Micro-Bypass Implantation for Primary Open-Angle Glaucoma Combined with Phacoemulsification: 4-Year Follow-Up. J Ophthalmol. 2015;2015:795357
13. Fea AM, Ahmed II, Lavia C, Mittica P, Consolandi G, Motolese I, et al. Hydrus microstent compared to selective laser trabeculoplasty in primary open angle glaucoma: one year results. Clin Exp Ophthalmol. 2017;45(2):120-127.
14. Fernández-Barrientos Y, García-Feijoó J, Martínez-de-la-Casa JM, Pablo LE, Fernández-Pérez C, García Sánchez J. Fluorophotometric study of the effect of the glaukos trabecular microbypass stent on aqueous humor dynamics. Invest Ophthalmol Vis Sci. 2010;51(7):3327-3332.
15. Gandolfi SA, Ungaro N, Ghirardini S, Tardini MG, Mora P. Comparison of Surgical Outcomes between Canaloplasty and Schlemm's Canal Scaffold at 24 Months' Follow-Up. J Ophthalmol. 2016;2016:3410469.
16. García-Feijoo J, Rau M, Grisanti S, Grisanti S, Höh H, Erb C, et al. Supraciliary Micro-stent Implantation for Open-Angle Glaucoma Failing Topical Therapy: 1-Year Results of a Multicenter Study. Am J Ophthalmol. 2015;159(6):1075-1081.
17. Gonnermann J, Bertelmann E, Pahlitzsch M, Maier AK, Torun N, Klamann MK. Contralateral eye comparison study in MICS & MIGS: Trabectome® vs. iStent inject®. Graefes Arch Clin Exp Ophthalmol. 2017;255(2):359-365.
18. Katz LJ, Erb C, Carceller GA, Fea AM, Voskanyan L, Wells JM, et al. Prospective, randomized study of one, two, or three trabecular bypass stents in open-angle glaucoma subjects on topical hypotensive medication. Clin Ophthalmol. 2015;9:2313-2320.
19. Khan M, Saheb H, Neelakantan A, Fellman R, Vest Z, Harasymowycz P, et al. Efficacy and safety of combined cataract surgery with 2 trabecular microbypass stents versus ab interno trabeculotomy. J Cataract Refract Surg. 2015;41(8):1716-1724
20. Klamann MK, Gonnermann J, Maier AK, Ruokonen PC, Torun N, Joussen AM, et al. Combined clear cornea phacoemulsification in the treatment of pseudoexfoliative glaucoma associated with cataract: significance of trabecular aspiration and ab interno trabeculectomy. Graefes Arch Clin Exp Ophthalmol. 2013;251(9):2195-2199
21. Kurji K, Rudnisky CJ, Rayat JS, Arora S, Sandhu S, Damji KF, et al. Phaco-trabectome versus phaco-iStent in patients with open-angle glaucoma. Can J Ophthalmol. 2017;52(1):99-106.
22. Lindstrom R, Lewis R, Hornbeak DM, Voskanyan L, Giamporcaro JE, Hovanesian J, et al. Outcomes Following Implantation of Two Second-Generation Trabecular Micro-Bypass Stents in Patients with Open-Angle Glaucoma on One Medication: 18-Month Follow-Up. Adv Ther. 2016;33(11):2082-2090.
23. Pahlitzsch M, Gonnermann J, Maier AK, Torun N, Bertelmann E, Joussen AM, et al. Trabeculectomy Ab Interno in Primary Open Angle Glaucoma and Exfoliative Glaucoma. Klin Monbl Augenheilkd. 2015;232(10):1198-1207.
24. Pahlitzsch M, Gonnermann J, Maier AK, Bertelmann E, Klamann MK, Erb C. Modified goniotomy as an alternative to trabectome in primary open angle glaucoma and pseudoexfoliation glaucoma: 1 year results. Can J Ophthalmol. 2017;52(1):92-98.
25. Pérez-Torregrosa VT, Olate-Pérez Á, Cerdà-Ibáñez M, Gargallo-Benedicto A, Osorio-Alayo V, Barreiro-Rego A, et al. Combined phacoemulsification and XEN45 surgery from a temporal approach and 2 incisions. Arch Soc Esp Oftalmol. 2016;91(9):415-421
26. Pfeiffer N, Garcia-Feijoo J, Martinez-de-la-Casa JM, Larrosa JM, Fea A, Lemij H, et al. A Randomized Trial of a Schlemm's Canal Microstent with Phacoemulsification for Reducing Intraocular Pressure in Open-Angle Glaucoma. Ophthalmology. 2015;122(7):1283-1293
27. Samuelson TW, Katz LJ, Wells JM, Duh YJ, Giamporcaro JE; US iStent Study Group. Randomized evaluation of the trabecular micro-bypass stent with phacoemulsification in patients with glaucoma and cataract. Ophthalmology. 2011 Mar;118(3):459-467
28. Spiegel D, Wetzel W, Neuhann T, Stuermer J, Hoeh H, Garcia-Feijoo J, et al. Coexistent primary open-angle glaucoma and cataract: interim analysis of a trabecular micro-bypass stent and concurrent cataract surgery. Eur J Ophthalmol. 2009;19(3):393-399
29. Ting JL, Damji KF, Stiles MC; Trabectome Study Group. Ab interno trabeculectomy: outcomes in exfoliation versus primary open-angle glaucoma. J Cataract Refract Surg. 2012;38(2):315-323
30. Töteberg-Harms M, Hanson JV, Funk J. Cataract surgery combined with excimer laser trabeculotomy to lower intraocular pressure: effectiveness dependent on preoperative IOP. BMC Ophthalmol. 2013;13:24.
31. Vold S, Ahmed II, Craven ER, Mattox C, Stamper R, Packer M, et al. Two-Year COMPASS Trial Results: Supraciliary Microstenting with Phacoemulsification in Patients with Open-Angle Glaucoma and Cataracts. Ophthalmology. 2016;123(10):2103-2112.
32. Vold SD, Voskanyan L, Tetz M, Auffarth G, Masood I, Au L, et al. Newly Diagnosed Primary Open-Angle Glaucoma Randomized to 2 Trabecular Bypass Stents or Prostaglandin: Outcomes Through 36 Months. Ophthalmol Ther. 2016;5(2):161-172.
33. Voskanyan L, García-Feijoó J, Belda JI, Fea A, Jünemann A, Baudouin C; Synergy Study Group. Prospective, unmasked evaluation of the iStent® inject system for open-angle glaucoma: synergy trial. Adv Ther. 2014;31(2):189-201
