# Supplementary material for: Minimally-invasive glaucoma surgeries (MIGS) for open angle glaucoma: A systematic review and meta-analysis
Source: PLoS One. 2017 Aug 29;12(8):e0183142. doi: 10.1371/journal.pone.0183142 (PMC5574616; doi:10.1371/journal.pone.0183142)
Supplement: S1 Table — (DOCX) [file pone.0183142.s003.docx]

**S1 Table. Risk of bias summary for Randomized Clinical Trials (RCTs): review authors’ judgements about each risk of bias item for each included study.**

| **BABIGHIAN 2010**^6^ |  |  |
| --- | --- | --- |
| **Bias** | **Authors' judgement** | **Support for judgement** |
| RANDOM SEQUENCE GENERATION (SELECTION BIAS) | LOW | QUOTE: "The randomization schedule and sequential numbering was generated using SAS version 8.2 (SAS Institute, Cary, NC, USA) program and stored in a locked cabinet." |
| ALLOCATION CONCEALMENT  (SELECTION BIAS) | UNCLEAR | COMMENT: randomization schedule and sequential numbering generator were stored in a locked cabinet. |
| BLINDING OF PARTICIPANTS, PERSONNNEL AND OUTCOME ASSESSORS (PERFORMANCE AND DETECTION BIAS) | LOW | QUOTE: “Authors evaluating the patients at each follow-up were blinded to whether the patient had undergone ELT or SLT .” |
| INCOMPLETE OUTCOME DATA  (ATTRITION BIAS) | LOW | COMMENT: no missing outcome data |
| SELECTIVE REPORTING  (REPORTING BIAS) | LOW | COMMENT: No study protocol is mentioned. However, all outcomes stated in the methods are reported in the results |
| OTHER BIAS | LOW | COMMENT: no conflict of interest declared |
|  |  |  |
| **CRAVEN 2012**^8^ (S=data from Samuelson 2011^27^, C=data from Craven 2012) | | |
| **Bias** | **Authors' judgement** | **Support for judgement** |
| RANDOM SEQUENCE GENERATION (SELECTION BIAS) | LOW | QUOTE: "He/she was assigned treatment according to a computer-generated randomization schedule (PROC PLAN, PC-SAS, SAS Inc., Cary NC) and scheduled for surgery" (S) |
| ALLOCATION CONCEALMENT  (SELECTION BIAS) | UNCLEAR | COMMENT: not reported |
| BLINDING OF PARTICIPANTS, PERSONNNEL AND OUTCOME ASSESSORS (PERFORMANCE AND DETECTION BIAS) | LOW | QUOTE: "Although the procedure was not masked, there is no way to see or identify the iStent at the slit lamp without gonioscopy; thus, the examiner was unable to tell to which group the patient belonged at the time of tonometry” (S)  “IOP was measured via applanation tonometry using a 2-person method in which 1 person performed the mesurement and 1 person recorded the value" (C) |
| INCOMPLETE OUTCOME DATA  (ATTRITION BIAS) | LOW | QUOTE: "Of the 5 subjects not examined at 12 months [...] in each case was deemed by the investigator to be unrelated to the treatment." (S)  COMMENT: reasons for missing data unlikely to be related to true outcome |
| SELECTIVE REPORTING  (REPORTING BIAS) | LOW | COMMENT: a study protocol is present. All outcomes stated in the methods are reported in the results |
| OTHER BIAS | UNCLEAR | COMMENT: funded by device industry, all authors affiliated with industry |
|  |  |  |
| **FEA 2014**^11^ |  |  |
| **Bias** | **Authors' judgement** | **Support for judgement** |
| RANDOM SEQUENCE GENERATION (SELECTION BIAS) | UNCLEAR | COMMENT: insufficient information about the sequence generation |
| ALLOCATION CONCEALMENT (SELECTION BIAS) | UNCLEAR | COMMENT: insufficient information |
| BLINDING OF PARTICIPANTS, PERSONNNEL AND OUTCOME ASSESSORS (PERFORMANCE AND DETECTION BIAS) | LOW | QUOTE: "It was not a masked study. These limitations are highly unlikely to have altered the findings that two iStent inject devices provide comparable benefits to combination medical therapy for OAG subjects" |
| INCOMPLETE OUTCOME DATA (ATTRITION BIAS) | UNCLEAR | COMMENT: insufficient information |
| SELECTIVE REPORTING  (REPORTING BIAS) | LOW | COMMENT: a study protocol is present. All outcomes stated in the methods are reported in the results |
| OTHER BIAS | UNCLEAR | COMMENT: funded by device industry, all authors affiliated with industry |
|  |  |  |
| **FEA 2015**^12^ (F1=data from Fea 2015, F2=data from Fea 2010^10^) | | |
| **Bias** | **Authors' judgement** | **Support for judgement** |
| RANDOM SEQUENCE GENERATION (SELECTION BIAS) | LOW | QUOTE: "Patient randomization was generated with a 2:1 ratio using Stata data analysis and statistical software (version 10, StataCorp LP)." (F2) |
| ALLOCATION CONCEALMENT (SELECTION BIAS) | UNCLEAR | COMMENT: not reported |
| BLINDING OF PARTICIPANTS, PERSONNNEL AND OUTCOME ASSESSORS (PERFORMANCE AND DETECTION BIAS) | UNCLEAR | QUOTE: "Patients were masked to their assignment, as were staff members who measured IOP throughout the study." (F2)  COMMENT: defined as open-label study |
| INCOMPLETE OUTCOME DATA (ATTRITION BIAS) | HIGH | COMMENT: imbalance in number for missing data (16.7% versus 41.7%) |
| SELECTIVE REPORTING  (REPORTING BIAS) | LOW | COMMENT: a study protocol is present. All outcomes stated in the methods are reported in the results |
| OTHER BIAS | LOW | COMMENT: no conflict of interest declared |
|  |  |  |
| **FERNÁNDEZ-BARRIENTOS 2010**^14^ |  |  |
| **Bias** | **Authors' judgement** | **Support for judgement** |
| RANDOM SEQUENCE GENERATION (SELECTION BIAS) | LOW | QUOTE: "Eliglible patients were randomly assigned to one of the two treatment groups with a computer-generated sequence" |
| ALLOCATION CONCEALMENT  (SELECTION BIAS) | UNCLEAR | COMMENT: not reported |
| BLINDING OF PARTICIPANTS, PERSONNNEL AND OUTCOME ASSESSORS (PERFORMANCE AND DETECTION BIAS) | UNCLEAR | QUOTE: "All postoperative evaluations, except for the gonioscopies/goniophotographs (JMC), were performed by the same examiner (YFB), who was masked to the type of surgery performed. "  COMMENT: defined as open-label study |
| INCOMPLETE OUTCOME DATA  (ATTRITION BIAS) | LOW | COMMENT: no missing outcome data on IOP and number of medications |
| SELECTIVE REPORTING  (REPORTING BIAS) | UNCLEAR | COMMENT: a study protocol is present but somehow differs from the reported study; in particular, flouorophotometry (reported as a principal objective in the study, was not mentioned in the protocol). Outcomes about adverse events incomplete |
| OTHER BIAS | UNCLEAR | COMMENT: funded by device industry, all authors affiliated with industry |
|  |  |  |
| **KATZ 2015**^18^ |  |  |
| **Bias** | **Authors' judgement** | **Support for judgement** |
| RANDOM SEQUENCE GENERATION (SELECTION BIAS) | LOW | QUOTE: "Randomization treatment assignments were generated using a pseudorandom number generator" |
| ALLOCATION CONCEALMENT (SELECTION BIAS) | UNCLEAR | COMMENT: not reported |
| BLINDING OF PARTICIPANTS, PERSONNNEL AND OUTCOME ASSESSORS (PERFORMANCE AND DETECTION BIAS) | HIGH | QUOTE: "Lack of masking to the study-treatment groups" |
| INCOMPLETE OUTCOME DATA (ATTRITION BIAS) | LOW | QUOTE: "Analyses of mean IOP at each postoperative exam were conducted on the number of available eyes at each exam that had not undergone secondary surgical interventions that might confound results.  COMMENT: reasons for missing outcome data unlikely to be related to true outcome. |
| SELECTIVE REPORTING  (REPORTING BIAS) | LOW | COMMENT: a study protocol is present. All outcomes stated in the protocol are reported in the results, although postoperative CCT not reported |
| OTHER BIAS | UNCLEAR | COMMENT: funded by device industry, all authors affiliated with industry |
|  |  |  |
| **PFEIFFER 2015**^26^ |  |  |
| **Bias** | **Authors' judgement** | **Support for judgement** |
| RANDOM SEQUENCE GENERATION (SELECTION BIAS) | LOW | QUOTE: "Patients […] were assigned randomly in a 1:1 ratio according to a computer-generated listing." |
| ALLOCATION CONCEALMENT (SELECTION BIAS) | LOW | QUOTE: "Listing was made just before surgery to undergo either combined or CS alone surgery" |
| BLINDING OF PARTICIPANTS, PERSONNNEL AND OUTCOME ASSESSORS (PERFORMANCE AND DETECTION BIAS) | LOW | QUOTE: "The tonometry protocol used a 2-person system (an observer and a reader)”.  COMMENT: Only patients were masked to treatment. However, IOP was measured as described above and the non-blinding of others unlikely to introduce bias. |
| INCOMPLETE OUTCOME DATA (ATTRITION BIAS) | LOW | COMMENT: by the end of the study, 2 patients in Hydrus and CS group and 5 patients in the CS group were lost to follow up. Reasons were not health-related and unlikely to be related to true outcome |
| SELECTIVE REPORTING  (REPORTING BIAS) | LOW | COMMENT: a study protocol is present. All outcomes stated in the methods are reported in the results |
| OTHER BIAS | UNCLEAR | COMMENT: funded by device industry, all authors affiliated with industry |
|  |  |  |
| **VOLD 2016^31^ (CyPass)** |  |  |
| **Bias** | **Authors' judgement** | **Support for judgement** |
| RANDOM SEQUENCE GENERATION (SELECTION BIAS) | UNCLEAR | COMMENT: insufficient information about the sequence generation |
| ALLOCATION CONCEALMENT (SELECTION BIAS) | UNCLEAR | COMMENT: Not reported |
| BLINDING OF PARTICIPANTS, PERSONNNEL AND OUTCOME ASSESSORS (PERFORMANCE AND DETECTION BIAS) | LOW | QUOTE: subjects did remain masked to treatment group throughout follow-up, and IOPs were recorded by masked technicians |
| INCOMPLETE OUTCOME DATA (ATTRITION BIAS) | UNCLEAR | COMMENT: insufficient information on patients at 24-months follow-up |
| SELECTIVE REPORTING  (REPORTING BIAS) | LOW | COMMENT: a study protocol is present. All outcomes stated in the methods are reported in the results |
| OTHER BIAS | UNCLEAR | COMMENT: Financial support received by some authors |
|  |  |  |
| **VOLD 2016^32^ (iStent)** |  |  |
| **Bias** | **Authors' judgement** | **Support for judgement** |
| RANDOM SEQUENCE GENERATION (SELECTION BIAS) | UNCLEAR | COMMENT: insufficient information |
| ALLOCATION CONCEALMENT (SELECTION BIAS) | UNCLEAR | COMMENT: Not reported |
| BLINDING OF PARTICIPANTS, PERSONNNEL AND OUTCOME ASSESSORS (PERFORMANCE AND DETECTION BIAS) | HIGH | COMMENT: Open label study |
| INCOMPLETE OUTCOME DATA (ATTRITION BIAS) | UNCLEAR | COMMENT: Almost all (2-Stent: 53, Travoprost: 47) patients followed at 12 months; 34 and 33 patients followed at 36 months but causes of missingness not reported |
| SELECTIVE REPORTING  (REPORTING BIAS) | LOW | COMMENT: a study protocol is present. All outcomes stated in the methods are reported in the results. |
| OTHER BIAS | UNCLEAR | COMMENT: Financial support received by some authors |
