# Supplementary material for: Minimally-invasive glaucoma surgeries (MIGS) for open angle glaucoma: A systematic review and meta-analysis
Source: PLoS One. 2017 Aug 29;12(8):e0183142. doi: 10.1371/journal.pone.0183142 (PMC5574616; doi:10.1371/journal.pone.0183142)
Supplement: S2 Table — (DOCX) [file pone.0183142.s004.docx]

**S2 Table. Risk of bias summary for non-RCTs Non-randomized Study of Intervention (NRS): review authors’ judgements about each risk of bias item for each included study.**

| **STUDY TYPE: NRS** | | | | | | | |
| --- | --- | --- | --- | --- | --- | --- | --- |
| **AUTHOR (YEAR)** | **BIAS DUE TO CONFOUNDING** | **BIAS IN SELECTION OF PARTICIPANTS INTO STUDY** | **BIAS IN CLASSIFICATION OF INTERVENTIONS** | **BIAS DUE TO DEVIATIONS FROM INTENDED INTERVENTION** | **BIAS DUE TO MISSING DATA** | **BIAS IN MEASUREMENT OF OUTCOMES** | **BIAS IN SELECTION OF THE REPORTED RESULT** |
| BABIGHIAN 2006^5^ | SERIOUS  Not adjusted for baseline IOP and glaucoma medication use. Glaucoma diagnosis was necessary in at least one eye. The eye with the higher IOP was treated with ELT. Serious confounding bias likely to exist. | LOW  Consecutive patients were included. | LOW  MIGS is a well-defined once-only surgical intervention | SERIOUS  Co-interventions (glaucoma medications) were not balanced across intervention groups | LOW  No losses to FU at 12 months | MODERATE  No masking strategy specified | LOW  Mean IOP is a well-defined measure |
| FEA 2016^13^ | MODERATE  Propensity Score was calculated based on relevant baseline variables (no significant differences between groups were observed) | LOW  Consecutive patients were included | LOW  MIGS and SLT are well-defined interventions | MODERATE  Co-intervention balanced across intervention groups (ocular hypotensive medications reintroduced in both groups basing on IOP and predefined target IOP) | LOW  1 patient was lost to follow up in the Hydrus group | MODERATE  No masking strategy specified | LOW  Mean IOP is a well-defined measure |
| GANDOLFI 2016^15^ | SERIOUS  Not adjusted for baseline variables (e.g. IOP, glaucoma medication use, OAG subtype) | SERIOUS  To be included in the study, patients had to be present at 24 months follow-up | LOW  MIGS and canaloplasty are well-defined once-only surgical interventions | SERIOUS  Co-interventions were not balanced across intervention groups | LOW  No losses to FU | MODERATE  No masking strategy specified | LOW  Mean IOP is a well-defined measure |
| GONNERMANN 2016^17^ | SERIOUS  Not adjusted for baseline variables (e.g. IOP, glaucoma medication use, OAG subtype) | SERIOUS  To be included in the study, patients had to be present at every follow-up examination | LOW  MIGS is a well-defined once-only surgical intervention | SERIOUS  Co-interventions were not balanced across intervention groups | LOW  Proportions of (7%) and reasons for missing participants were similar across intervention groups | MODERATE  No masking strategy specified | SERIOUS  Mean IOP is a well-defined measure  Patients receiving secondary surgery excluded from the efficacy analysis |
| KHAN 2015^19^ | SERIOUS  Not adjusted for baseline variables (e.g. IOP, glaucoma medication use, OAG subtype and glaucoma severity). | SERIOUS  To be included in the study, patients had to be present at 12 months follow-up | LOW  MIGS is a well-defined once-only surgical intervention | SERIOUS  Co-interventions were not balanced across intervention groups | LOW  No losses to FU at 12 months | MODERATE  No masking strategy specified | LOW  Mean IOP is a well-defined measure |
| KLAMANN 2013^20^ | SERIOUS  Not adjusted for baseline variables (e.g. IOP, glaucoma medication use) | LOW  Subject were recruited consecutively | LOW  MIGS is a well-defined once-only surgical intervention | MODERATE  Co-interventions were balanced across intervention groups (“glaucoma medications were continued” ... “to keep the target pressure lower than 16 mmHg in both groups”) | LOW  No losses to FU at 12 months | MODERATE  No masking strategy specified | LOW  Mean IOP is a well-defined measure |
| KURJI 2016^21^ | SERIOUS  Not adjusted for baseline variables (e.g. IOP, glaucoma medication use, OAG subtype) | LOW  Subject were recruited consecutively | LOW  MIGS is a well-defined once-only surgical intervention | SERIOUS  Co-interventions were not balanced across intervention groups | LOW  3 patients were lost to follow up in each group | MODERATE  No masking strategy specified | LOW  Mean IOP is a well-defined measure |
| PAHLITZSCH 2016^24^ | SERIOUS  Not adjusted for baseline variables (e.g. IOP, glaucoma medication use) | SERIOUS  To be included in the study, patients had to be present at all follow-up visits | LOW  MIGS are well-defined once-only surgical interventions | MODERATE  Co-interventions were balanced across intervention groups (“criteria to reintroduce medication included individual IOP measurements above the target IOP”) | LOW  No losses to FU at 12 months | MODERATE  No masking strategy specified | NO INFORMATION  Mean IOP is a well-defined measure. It is not specified if the efficacy results included patients who underwent additional surgery during the follow-up |
