# Supplementary material for: Minimally-invasive glaucoma surgeries (MIGS) for open angle glaucoma: A systematic review and meta-analysis
Source: PLoS One. 2017 Aug 29;12(8):e0183142. doi: 10.1371/journal.pone.0183142 (PMC5574616; doi:10.1371/journal.pone.0183142)
Supplement: S3 Table — (DOCX) [file pone.0183142.s005.docx]

**S3 Table. Risk of bias summary for non-RCTs Before-after studies: review authors’ judgements about each risk of bias item for each included study.**

| **STUDY TYPE: BEFORE-AFTER** | | | | | | | |
| --- | --- | --- | --- | --- | --- | --- | --- |
| **AUTHOR (YEAR)** | **BIAS DUE TO CONFOUNDING** | **BIAS IN SELECTION OF PARTICIPANTS INTO STUDY** | **BIAS IN CLASSIFICATION OF INTERVENTIONS** | **BIAS DUE TO DEVIATIONS FROM INTENDED INTERVENTION** | **BIAS DUE TO MISSING DATA** | **BIAS IN MEASUREMENT OF OUTCOMES** | **BIAS IN SELECTION OF THE REPORTED RESULT** |
| AHMED 2014^1^ | NA  Confounding domain not applicable to before-after studies | NO INFORMATION | LOW  MIGS is a well-defined once-only surgical intervention | LOW  IOP was measured after wash-out | LOW  No losses to FU at 12 months | MODERATE  No masking strategy specified | LOW  Mean IOP is a well-defined measure |
| ARRIOLA-VILLALOBOS 2012^2^ | NA  Confounding domain not applicable to before-after studies | NO INFORMATION | LOW  MIGS is a well-defined once-only surgical intervention | SERIOUS  IOP was NOT measured after wash-out | LOW  No losses to FU at 12 months | MODERATE  No masking strategy specified | LOW  Mean IOP is a well-defined measure |
| ARRIOLA-VILLALOBOS 2016^4^ | NA  Confounding domain not applicable to before-after studies | LOW  Subject were recruited consecutively (as specified in Arriola-Villalobos 2013^3^) | LOW  MIGS is a well-defined once-only surgical intervention | MODERATE  IOP was measured after wash-out but only at baseline | LOW  No losses to FU at 12 months | MODERATE  No masking strategy specified | LOW  Mean IOP is a well-defined measure |
| BABIGHIAN 2006^5^ | NA  Confounding domain not applicable to before-after studies | LOW  Consecutive patients were included. | LOW  MIGS is a well-defined once-only surgical intervention | SERIOUS  IOP was NOT measured after wash-out | LOW  No losses to FU at 12 months | MODERATE  No masking strategy specified | LOW  Mean IOP is a well-defined measure |
| BABIGHIAN 2010^6^ | NA  Confounding domain not applicable to before-after studies | LOW  Consecutive patients were included. | LOW  MIGS is a well-defined once-only surgical intervention | SERIOUS  IOP was NOT measured after wash-out | LOW  No losses to FU at 12 months | MODERATE  Authors were blinded to intervention type but not to intervention status | LOW  Mean IOP is a well-defined measure |
| BELOVAY 2012^7^ | NA  Confounding domain not applicable to before-after studies | SERIOUS  To be included in the study, patients had to be present at every follow-up examination | LOW  MIGS is a well-defined once-only surgical intervention | SERIOUS  IOP was NOT measured after wash-out | LOW  1 patient was lost to 12 months follow-up in one group | MODERATE  No masking strategy specified | LOW  Mean IOP is a well-defined measure |
| CRAVEN 2012^8^ | NA  Confounding domain not applicable to before-after studies | LOW  Consecutive patients were included. | LOW  MIGS is a well-defined once-only surgical intervention | MODERATE  IOP was measured after wash-out but only at baseline | LOW  NO losses to FU at 12 months | MODERATE  No masking strategy | LOW  Mean IOP is a well-defined measure |
| DONNENFELD 2015^9^ | NA  Confounding domain not applicable to before-after studies | SERIOUS  Patients not meeting inclusion criteria were included in the per protocol population | MODERATE  MIGS is a well-defined once-only surgical intervention. “For many of the surgeons, this was their first experience implanting a MIGS device. The study group comprises visiting surgeons and one staff surgeon at one investigational site” | MODERATE  IOP was measured after wash-out but only at baseline | LOW  No losses to FU at 12 months | MODERATE  No masking strategy | LOW  Mean IOP is a well-defined measure |
| FEA 2014^11^ | NA  Confounding domain not applicable to before-after studies | NO INFORMATION | LOW  MIGS is a well-defined once-only surgical intervention | MODERATE  IOP was measured after wash-out but only at baseline | LOW  No losses to FU at 12 months | MODERATE  No masking strategy specified | LOW  Mean IOP is a well-defined measure |
| FEA 2015^12^ | NA  Confounding domain not applicable to before-after studies | NO INFORMATION | LOW  MIGS is a well-defined once-only surgical intervention | LOW  IOP was measured after wash-out | LOW  No losses to FU at 12 months | MODERATE  Unclear masking strategy specified | LOW  Mean IOP is a well-defined measure |
| FEA 2016^13^ | NA  Confounding domain not applicable to before-after studies | LOW  Consecutive patients were included | LOW  MIGS is a well-defined once-only surgical intervention | SERIOUS  IOP was not measured after wash-out | LOW  1 patient was lost to follow up in the Hydrus group | MODERATE  No masking strategy specified | LOW  Mean IOP is a well-defined measure |
| FERNÁNDEZ-BARRIENTOS 2010^14^ | NA  Confounding domain not applicable to before-after studies | NO INFORMATION | LOW  MIGS is a well-defined once-only surgical intervention | MODERATE  IOP was measured after wash-out but only at baseline | LOW  No losses to FU at 12 months | MODERATE  Authors were blinded to intervention type but not to intervention status | LOW  Mean IOP is a well-defined measure |
| GANDOLFI 2016^15^ | NA  Confounding domain not applicable to before-after studies | SERIOUS  To be included in the study, patients had to be present at 24 months follow-up | LOW  MIGS is a well-defined once-only surgical intervention | SERIOUS  IOP was not measured after wash-out | LOW  No losses to FU | MODERATE  No masking strategy specified | LOW  Mean IOP is a well-defined measure |
| GARCìA-FEIJOO 2015^16^ | NA  Confounding domain not applicable to before-after studies | NO INFORMATION | LOW  MIGS is a well-defined once-only surgical intervention | SERIOUS  IOP was not measured after wash-out | LOW  15% losses to follow-up at 12 months | MODERATE  No masking strategy specified | SERIOUS  Mean IOP is a well-defined measure  Patients receiving secondary surgery excluded from the efficacy analysis |
| GONNERMANN 2016^17^ (Trabectome and iStent) | NA  Confounding domain not applicable to before-after studies | SERIOUS  To be included in the study, patients had to be present at every follow-up examination | LOW  MIGS is a well-defined once-only surgical intervention | SERIOUS  IOP was not measured after wash-out | LOW  Proportions of (7%) and reasons for missing participants were similar across intervention groups | MODERATE  No masking strategy specified | SERIOUS  Mean IOP is a well-defined measure  Patients receiving secondary surgery excluded from the efficacy analysis |
| KATZ 2015  (one stent group, two stent group and 3 stent group) | NA  Confounding domain not applicable to before-after studies | NO INFORMATION | LOW  MIGS is a well-defined once-only surgical intervention | LOW  IOP was measured after wash-out | LOW  1 patient lost to follow-up in 1-stent-group, 2 patients in 2-stent group and none in 3-stent-group | MODERATE  No masking strategy specified | LOW  Mean IOP is a well-defined measure |
| KHAN 2015^19^ | NA  Confounding domain not applicable to before-after studies | SERIOUS  To be included in the study, patients had to be present at 12 months follow-up | LOW  MIGS is a well-defined once-only surgical intervention | SERIOUS  IOP was NOT measured after wash-out | LOW  No losses to FU at 12 months | MODERATE  No masking strategy specified | LOW  Mean IOP is a well-defined measure |
| KLAMANN 2013^20^ (Trabectome) | NA  Confounding domain not applicable to before-after studies | LOW  Subject were recruited consecutively | LOW  MIGS is a well-defined once-only surgical intervention | SERIOUS  IOP was NOT measured after wash-out | LOW  No losses to FU at 12 months | MODERATE  No masking strategy specified | LOW  Mean IOP is a well-defined measure |
| KURJI 2016^21^ (Trabectome and iStent group) | NA  Confounding domain not applicable to before-after studies | LOW  Subject were recruited consecutively | LOW  MIGS is a well-defined once-only surgical intervention | SERIOUS  IOP was NOT measured after wash-out | LOW  3 patients were lost to follow up in each group | MODERATE  No masking strategy specified | LOW  Mean IOP is a well-defined measure |
| LINDSTROM 2016^22^ | NA  Confounding domain not applicable to before-after studies | NO INFORMATION | LOW  MIGS is a well-defined once-only surgical intervention | MODERATE  IOP was measured after wash-out but only at baseline | LOW  No losses to FU at 12 months | MODERATE  No masking strategy | LOW  Mean IOP is a well-defined measure |
| PAHLITZSCH 2015^23^ (Trabectome solo and combined) | NA  Confounding domain not applicable to before-after studies | SERIOUS  To be included in the study, patients had to be present at every follow-up examination | LOW  MIGS is a well-defined once-only surgical intervention | SERIOUS  IOP was NOT measured after wash-out | LOW  No losses to FU at 12 months | MODERATE  No masking strategy | LOW  Mean IOP is a well-defined measure |
| PAHLITZSCH 2016^24^ | NA  Confounding domain not applicable to before-after studies | SERIOUS  To be included in the study, patients had to be present at all follow-up visits | LOW  MIGS are well-defined once-only surgical interventions | SERIOUS  IOP was NOT measured after wash-out | LOW  No losses to FU at 12 months | MODERATE  No masking strategy specified | NO INFORMATION  Mean IOP is a well-defined measure. It is not specified if the efficacy results included patients who underwent additional surgery during the follow-up |
| PÉREZ-TORREGROSA 2016^25^ | NA  Confounding domain not applicable to before-after studies | NO INFORMATION | LOW  MIGS is a well-defined once-only surgical intervention | SERIOUS  IOP was NOT measured after wash-out | LOW  No losses to FU at 12 months | MODERATE  No masking strategy specified | LOW  Mean IOP is a well-defined measure |
| PFEIFFER 2015^26^ | NA  Confounding domain not applicable to before-after studies | NO INFORMATION | LOW  MIGS are well-defined once-only surgical interventions | LOW  IOP was measured after wash-out | LOW  4% losses to FU at 12 months | MODERATE  No masking strategy | NO INFORMATION  Mean IOP is a well-defined measure |
| SPIEGEL 2009^28^ | NA  Confounding domain not applicable to before-after studies | NO INFORMATION | LOW  MIGS is a well-defined once-only surgical intervention | SERIOUS  IOP was NOT measured after wash-out | LOW  13% losses to follow-up at 12 months | MODERATE  No masking strategy | SERIOUS  Mean IOP is a well-defined measure. Patients receiving secondary surgery excluded from the efficacy analysis |
| TING 2012^29^ | NA  Confounding domain not applicable to before-after studies | SERIOUS  To be included in the study, patients had to be present at 12 months follow-up | LOW  MIGS is a well-defined once-only surgical intervention | SERIOUS  IOP was NOT measured after wash-out | LOW  6% losses to follow-up at 12 months | MODERATE  No masking strategy | LOW  Mean IOP is a well-defined measure |
| TÖTEBERG-HARMS 2013^30^ | NA  Confounding domain not applicable to before-after studies | MODERATE  Subject were recruited consecutively BUT If a patient received phaco-ELT in both eyes, only the first eye was considered. | LOW  MIGS is a well-defined once-only surgical intervention | SERIOUS  IOP was NOT measured after wash-out | LOW  No losses to FU at 12 months | MODERATE  No masking strategy specified | LOW  Mean IOP is a well-defined measure |
| VOLD 2016^31^ CyPass | NA  Confounding domain not applicable to before-after studies | NO INFORMATION | LOW  MIGS is a well-defined once-only surgical intervention | LOW  IOP was measured after wash-out | LOW  No losses to FU at 12 months | LOW  IOP was recorded by masked technicians | LOW  Mean IOP is a well-defined measure |
| VOLD 2016^32^ | NA  Confounding domain not applicable to before-after studies | NO INFORMATION | LOW  MIGS is a well-defined once-only surgical intervention | MODERATE  Naïve patients. Postoperatively, IOP was not measured after wash-out | LOW  2% losses to follow-up at 12 months | MODERATE  No masking strategy | LOW  Mean IOP is a well-defined measure |
| VOSKANYAN 2014^33^ | NA  Confounding domain not applicable to before-after studies | SERIOUS  Patients with postoperative IOP greater than 38 mmHg exited from the study | LOW  MIGS is a well-defined once-only surgical intervention | MODERATE  IOP was measured after wash-out but only at baseline. Mean diurnal IOP was recorded at selected sites | LOW  5% losses to follow-up at 12 months | MODERATE  No masking strategy | LOW  Mean IOP is a well-defined measure |
