# Supplementary material for: Minimally-invasive glaucoma surgeries (MIGS) for open angle glaucoma: A systematic review and meta-analysis
Source: PLoS One. 2017 Aug 29;12(8):e0183142. doi: 10.1371/journal.pone.0183142 (PMC5574616; doi:10.1371/journal.pone.0183142)
Supplement: S4 Table — (DOCX) [file pone.0183142.s006.docx]

**S4 Table. Risk of bias sponsor-related for all studies.**

| **AUTHOR, YEAR** | **STUDY DESIGN** | **COMMERCIAL RELATIONSHIP/FUNDING** |
| --- | --- | --- |
| AHMED 2014^1^ | BEF-AFT STUDY | ALL AUTHORS ARE CONSULTANTS. |
| ARRIOLA-VILLALOBOS 2012^2^ | BEF-AFT STUDY | ONE AUTHOR IS A CONSULTANT.  SPONSOR PROVIDED STUDY DEVICES. |
| ARRIOLA-VILLALOBOS 2016^4^ | BEF-AFT STUDY | ONE AUTHOR IS A CONSULTANT.  SPONSOR PROVIDED STUDY DEVICES. |
| BABIGHIAN 2006^5^ | NRS | NR |
| BABIGHIAN 2010^6^ | RCT | NO |
| BELOVAY 2012^7^ | NRS | ONE AUTHOR IS A CONSULTANT. |
| CRAVEN 2012^8^ | RCT | ALL AUTHORS ARE CONSULTANTS.  FUNDING WAS PROVIDED BY SPONSOR. |
| DONNENFELD 2015^9^ | BEF-AFTER STUDY | ALL AUTHORS ARE CONSULTANTS, SOME AUTHORS RECEIVED FINANCIAL SUPPORT.  SPONSOR PROVIDED STUDY DEVICES. |
| FEA 2014^11^ | RCT | SOME AUTHORS RECEIVED FINANCIAL SUPPORT.  SPONSOR PROVIDED STUDY DEVICES |
| FEA 2015^12^ | RCT | NO |
| FEA 2016^13^ | NRS | ONE AUTHOR IS A CONSULTANT. |
| FERNÁNDEZ-BARRIENTOS 2010^14^ | RCT | SOME AUTHORS ARE CONSULTANT AND/OR RECEIVED FINANCIAL SUPPORT.  THE STUDY WAS SUPPORTED BY SPONSOR. |
| GANDOLFI 2016^15^ | NRS | NO |
| GARCìA-FEIJOO 2015^16^ | BEF-AFT STUDY | AUTHORS ARE CONSULTANT AND/OR RECEIVED FINANCIAL SUPPORT.  FUNDING WAS PROVIDED BY SPONSOR. |
| GONNERMANN 2016^17^ | NRS | NO |
| KATZ 2015^18^ | RCT | SOME AUTHORS RECEIVED FINANCIAL SUPPORT.  FUNDING WAS PROVIDED BY SPONSOR. |
| KHAN 2015^19^ | NRS | SOME AUTHORS ARE CONSULTANT AND/OR RECEIVED FUNDING. |
| KLAMANN 2013^20^ | NRS | NO |
| KURJI 2016^21^ | NRS | NO |
| LINDSTROM 2016^22^ | BEF-AFT STUDY | SOME AUTHORS ARE CONSULTANT AND/OR RECEIVED FINANCIAL SUPPORT.  SPONSOR PROVIDED STUDY DEVICES. |
| PAHLITZSCH 2015^23^ | NRS | NO |
| PAHLITZSCH 2016^24^ | NRS | NO |
| PÉREZ-TORREGROSA 2016^25^ | BEF-AFT STUDY | NO |
| PFEIFFER 2015^26^ | RCT | AUTHORS ARE CONSULTANT AND/OR RECEIVED FINANCIAL SUPPORT.  STUDY FUNDING WAS PROVIDED BY SPONSOR. |
| SPIEGEL 2009^28^ | BEF-AFT STUDY | SOME AUTHORS ARE CONSULTANTS.  STUDY FUNDING WAS PROVIDED BY SPONSOR. |
| TING 2012^29^ | NRS | NO |
| TÖTEBERG-HARMS 2013^30^ | BEF-AFT STUDY | NO |
| VOLD 2016^31^ CyPass | RCT | AUTHORS ARE CONSULTANT AND/OR RECEIVED FINANCIAL SUPPORT. |
| VOLD 2016^32^ | RCT | AUTHORS RECEIVED FINANCIAL SUPPORT.  SPONSOR PROVIDED STUDY DEVICES. |
| VOSKANYAN 2014^33^ | BEF-AFT STUDY | ALL AUTHORS RECEIVED FINANCIAL SUPPORT.  SPONSOR PROVIDED STUDY DEVICES. |

NR=NOT-REPORTED
