# Supplementary material for: Minimally-invasive glaucoma surgeries (MIGS) for open angle glaucoma: A systematic review and meta-analysis
Source: PLoS One. 2017 Aug 29;12(8):e0183142. doi: 10.1371/journal.pone.0183142 (PMC5574616; doi:10.1371/journal.pone.0183142)

**S1 Figure. Risk of bias summary for RCTs: review authors’ judgements about each risk of bias item for each included study.**
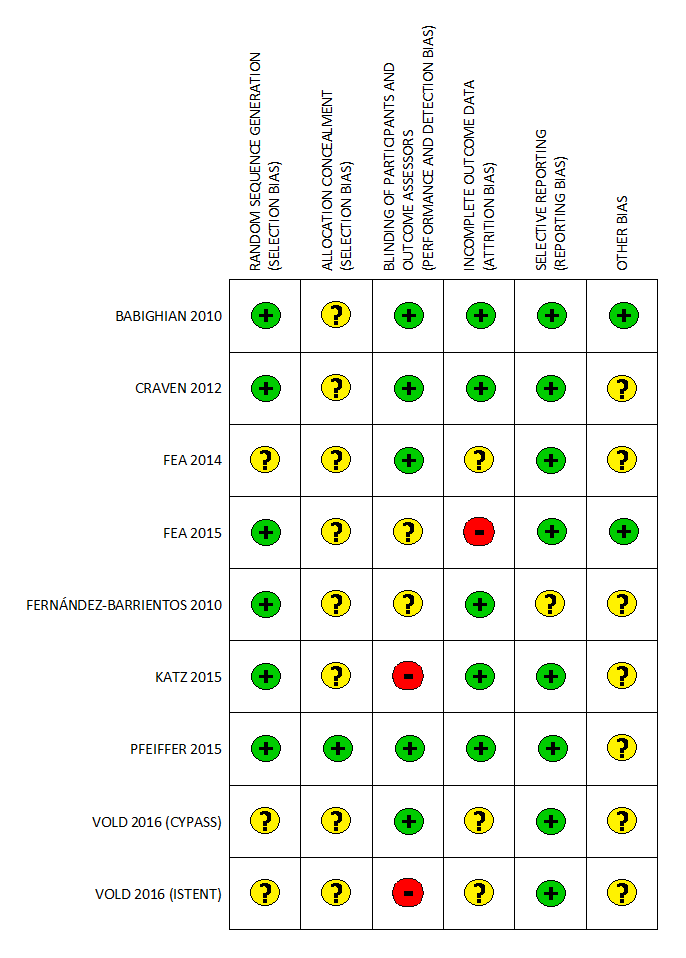

Supplement: S1 Fig — (DOCX) [file pone.0183142.s007.docx]
