# Supplementary material for: Minimally-invasive glaucoma surgeries (MIGS) for open angle glaucoma: A systematic review and meta-analysis
Source: PLoS One. 2017 Aug 29;12(8):e0183142. doi: 10.1371/journal.pone.0183142 (PMC5574616; doi:10.1371/journal.pone.0183142)
Supplement: S4 Fig — (DOCX) [file pone.0183142.s010.docx]

**S4 Figure. Methodological quality graph for non-RCTs (Before-after): review authors’ judgements about each methodological quality item of ROBINS-I presented as percentages across all included studies.**
