# Supplementary material for: Minimally-invasive glaucoma surgeries (MIGS) for open angle glaucoma: A systematic review and meta-analysis
Source: PLoS One. 2017 Aug 29;12(8):e0183142. doi: 10.1371/journal.pone.0183142 (PMC5574616; doi:10.1371/journal.pone.0183142)
Supplement: S6 Fig — Values expressed in Weighed Mean Difference (WMD). (DOCX) [file pone.0183142.s012.docx]

**S6 Figure. Forest plot for 24-months medication reduction**

Legend: Values expressed in Weighed Mean Difference (WMD).
